# Intranasal COVID-19 vaccine induces respiratory memory T cells and protects K18-hACE mice against SARS-CoV-2 infection

**DOI:** 10.1038/s41541-023-00665-3

**Published:** 2023-05-13

**Authors:** Béré K. Diallo, Caitlín Ní Chasaide, Ting Y. Wong, Pauline Schmitt, Katherine S. Lee, Kelly Weaver, Olivia Miller, Melissa Cooper, Seyed D. Jazayeri, F. Heath Damron, Kingston H. G. Mills

**Affiliations:** 1 Immune Regulation Research Group, School of Biochemistry and Immunology, Trinity Biomedical Sciences Institute, Trinity College Dublin, Dublin, Ireland; 2 Department of Microbiology, Immunology, and Cell Biology and Vaccine Development Center, West Virginia University, Health Sciences Center, Morgantown, West Virginia USA

**Keywords:** Vaccines, Viral infection

## Abstract

Current COVID-19 vaccines prevent severe disease, but do not induce mucosal immunity or prevent infection with SARS-CoV-2, especially with recent variants. Furthermore, serum antibody responses wane soon after immunization. We assessed the immunogenicity and protective efficacy of an experimental COVID-19 vaccine based on the SARS-CoV-2 Spike trimer formulated with a novel adjuvant LP-GMP, comprising TLR2 and STING agonists. We demonstrated that immunization of mice twice by the intranasal (i.n.) route or by heterologous intramuscular (i.m.) prime and i.n. boost with the Spike-LP-GMP vaccine generated potent Spike-specific IgG, IgA and tissue-resident memory (T_RM_) T cells in the lungs and nasal mucosa that persisted for at least 3 months. Furthermore, Spike-LP-GMP vaccine delivered by i.n./i.n., i.m./i.n., or i.m./i.m. routes protected human ACE-2 transgenic mice against respiratory infection and COVID-19-like disease following lethal challenge with ancestral or Delta strains of SARS-CoV-2. Our findings underscore the potential for nasal vaccines in preventing infection with SARS-CoV-2 and other respiratory pathogen.

## Introduction

First-generation mRNA and adenovirus-vectored COVID-19 vaccines were effective in controlling the COVID-19 pandemic early after their licensure. Phase 3 clinical trials showed that they had efficacies of between 60 and 95% against symptomatic COVID-19^1–5^. However, most of these trials did not assess protection against SARS-CoV-2 infection and real-world data has indicated that current vaccines had lower efficacy against asymptomatic COVID-19 and did not prevent transmission of the virus. Furthermore, with the emergence of variants of concern (VOC), their effectiveness was drastically reduced^6^. In addition, there is rapid waning of the immunity in vaccinated individuals, with a significant decline in serum antibodies against the SARS-CoV-2 Spike protein a few months after vaccination^7–9^.

Although SARS-CoV-2 can disseminate from the respiratory tract, it first infects individuals in the nasal mucosa. All first-generation COVID-19 vaccines are delivered via the parenteral (intramuscular) route, and this promotes strong systemic IgG and T cell responses, which confer a relatively high level of protection against severe COVID-19 disease. However, unlike natural infection with SARS-CoV-2, current vaccines do not induce mucosal IgA or respiratory T cell responses or prevent infection of the respiratory tract, especially against variants that have multiple mutations in the Spike protein^10,11^. The latter reflects escape from neutralizing antibodies generated against the historical strain of SARS-CoV-2^12–14^. In contrast, previous infection can prevent infection as well as symptomatic disease, especially against closely related viral variants; for example, infection with Omicron BA.1 confers 70–80% protection against infection with BA.4 or BA-5^15^. Therefore, there is a need to develop new COVID-19 vaccines that induce mucosal immunity and prevent respiratory infection against multiple strains of SARS-CoV-2 and that can also induce local immunological memory, analogous to that induced with natural infection.

Recent studies have shown that tissue-resident memory T (T_RM_) cells in the mucosal tissue mediate long-term protection against mucosal pathogens^16,17^. In SARS-CoV-2 infection, T_RM_ cells are induced in the lungs of patients with severe and mild COVID-19 and persist for up to 10 months, suggesting a role in the long-term protection of the mucosal tissue^18,19^. Our work on the respiratory bacterial pathogen *Bordetella pertussis* has shown that CD4^+^ T_RM_ cells confer sustained protective immunity in the lungs and nasal tissue induced by previous infection^20,21^. Furthermore, we demonstrated that nasal immunization with an acellular (subunit) pertussis vaccine formulated with the novel adjuvant LP-GMP also generated persistent sterilizing immunity in the upper and lower respiratory tract^20^. LP-GMP is a combination of LP1569, a synthetic lipopeptide TLR2 agonist^22^ and C-di-GMP a synthetic STING agonist^23^. TLR2 activation by LP1569 delivers a signal through MyD88 pathway to activate NF-κB and AP1, leading to transcription of inflammatory cytokines. STING directly senses C-di-GMP, leading to downstream activation of the IRF3 and NF-κB, which induces production of type I interferons, IFN-stimulated genes (ISGs) and inflammatory cytokines^24^. The combination of LP1569 and C-di-GMP synergistically promotes IL-12, IL-23, IL-1β, TNF and IL-6 production by dendritic cells. This combination of inflammatory cytokines promotes the induction of Th1 and Th17 cells. A pertussis vaccine delivered by nasal route containing LP-GMP as the adjuvant promoted IgG, IgA and IFN-γ and IL-17-secreting T_RM_ cells in the lungs and nasal tissue^20^.

Intranasal immunization is an effective route for generating strong mucosal immunity with COVID-19 vaccines^25,26^. However, most studies on intranasal COVID-19 vaccines have focused on antibody and T cells responses in the lungs, rather than the nasal mucosa, which is the first site of infection after exposure to SARS-CoV-2. The generation of potent antibody and T cell responses in the nose is key to inducing sterilizing immunity as well as local immunological memory against SARS-CoV-2. In this study, we evaluated the immunogenicity and protective efficacy of an experimental COVID-19 vaccine comprising the SARS-CoV-2 Spike trimer adjuvanted with the TLR2 agonist, LP1569, and the STING agonist, C-di-GMP, named Spike-LP-GMP. We demonstrated that intranasal immunization with Spike-LP-GMP elicits systemic Spike-specific IgG and T cells. More importantly, we found substantial Spike-specific IgG, IgA and T_RM_ cells that secrete IFN-γ, TNF, IL-17 and Granzyme B (GrB) in the nasal mucosa as well as the lungs. Finally, we showed that intranasal (i.n.) immunization or intramuscular (i.m.) priming followed by i.n. boosting with Spike-LP-GMP protected K18-hACE-2 against a lethal challenge with SARS-CoV-2 Washington (WA-1; Wuhan-like) or Delta strains and significantly reduced the viral RNA burden in the respiratory tract and brain.

## Results

### An intranasally-delivered SARS-CoV-2 vaccine with LP-GMP as the adjuvant induces local humoral and cellular immunity in the lungs and nasal mucosa

We previously demonstrated that LP-GMP, which combines TLR2 and STING agonists, is a potent adjuvant when used in a novel pertussis vaccine, and conferred sterilizing immunity in the nasal mucosa, especially when delivered by the i.n. route^20^. Since nasally delivered vaccines with a mutant of *Escherichia* coli heat labile toxin as the adjuvant has been associated with transient facial nerve paralysis^27^, we first assessed the potential reactogenicity of LP-GMP. We had previously demonstrated that unlike cholera toxin, LP-GMP did not induce IL-1β or TNF in the olfactory bulb or hypothalamus after i.n. admisntration^20^. Here we assessed local and systemic inflammatory responses induced with LP-GMP compared with LPS. Mice were immunized i.n. with Spike-LP-GMP, Spike-LPS or PBS and inflammatory cytokines were assessed in the nasal tissue, lungs, and serum 24 and 72 h after administration. While LPS promoted production of IL-6, IL-1β and TNF in the nasal tissue, lungs and serum of mice, the Spike-LP-GMP experimental vaccine did not significantly increase inflammatory cytokine production over that detected in mice immunized with PBS (supplementary Fig. 1).

**Fig. 1:**
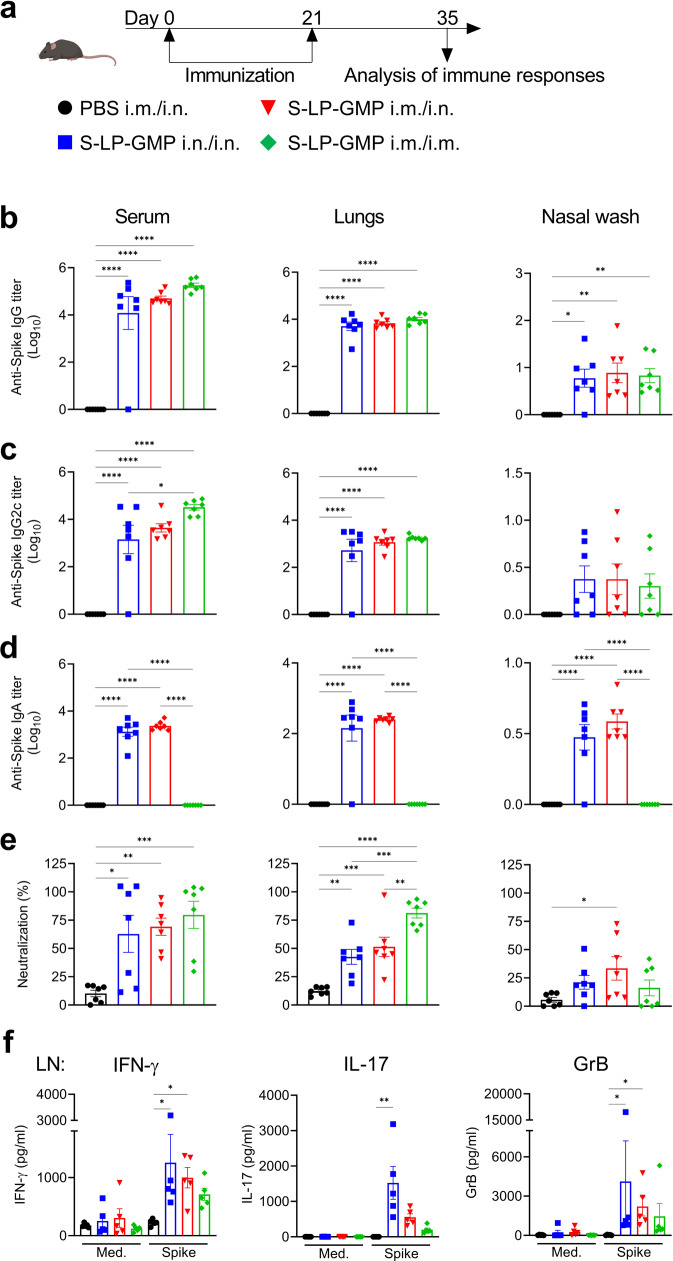
The Spike-LP-GMP vaccine delivered by i.n. and/or i.m. routes induces potent antibodies and peripheral T cell responses. **a** Schematic of experimental design; C57BL/6 mice (*n* = 7/group) were immunized twice (0 and 21 days) i.n./i.n., i.m./i.n. or i.m./i.m. with the Spike-LP-GMP vaccine or PBS and euthanized on day 35 for samples collection. Spike-specific IgG **b**, IgG2c **c**, and IgA **d**, and hACE-2-Spike neutralizing antibodies **e** in the serum, lung homogenate and nasal wash. **f** Antigen-specific IFN-γ, IL-17 and GrB production by cervical and inguinal LN cells stimulated for 72 h with medium alone or Spike trimer (2.5 µg/ml), quantified by ELISA. Data were analyzed by one-way ANOVAs followed by post hoc Tukey’s test for multiple comparisons (*P < 0.05, **P < 0.01, ***P < 0.001, and ****P < 0.0001), error bars, SEM. Results are representative of three independent experiments.

Next, we examined if LP-GMP could be an effective adjuvant for enhancing the immunogenicity of the SARS-CoV-2 Spike trimer delivered intranasally. Mice were immunized with a vaccine formulation containing the Spike trimer (Wuhan strain) and LP-GMP (henceforth referred to as Spike-LP-GMP) using parenteral (i.m./i.m.), mucosal (i.n./i.n.) or heterologous prime-boost (i.m./i.n.) approaches. C57BL/6 mice were primed on day 0 and boosted on day 21, and immune responses were assessed on day 35 (Fig. 1a). We found that each of the three immunization strategies induced Spike-specific IgG, including IgG2c in the serum, lung homogenate and nasal wash (Fig. 1b, c). However, only the i.n./i.n. or i.m./i.n. immunization approaches induced IgA in the serum, lung and nasal cavity. A single i.n. immunization after parenteral priming induced IgA in the upper and lower respiratory tract of mice, whereas two immunizations by the i.m. route failed to induce mucosal IgA responses (Fig. 1d). Furthermore, our experimental vaccine induced Spike-specific secretory IgA in the lungs and nasal mucosa when delivered by the i.n./i.n. or i.m./i.n., but not i.m./i.m., routes (Supplementary Fig. 2a). All three immunization approaches elicited virus neutralizing antibodies in serum and lung assessed, using an assay based on the inhibition of Spike-ACE-2 interaction (Fig. 1e).

We examined antigen-specific systemic T cell responses induced with the Spike-LP-GMP vaccine by restimulation of draining lymph nodes (LN) cells with the Spike trimer. Antigen-specific IFN-γ, IL-17 and granzyme B (GrB) were produced by LN mice immunized with Spike-LP-GMP by all 3 immunization approaches, but the responses were most potent following immunization by i.n./i.n. or i.n./i.m. routes (Fig. 1f). In addition, the i.n./i.n. route of immunization induced Spike-specific IFN-γ, IL-17 and GrB in the cervical LN and the inguinal LN (Supplementary Fig. 2b, c). In contrast, the i.m./i.m. route of immunization induced Spike-specific IFN-γ and GrB only in the inguinal LN (Supplementary Fig. 2c). These findings demonstrated that the Spike-LP-GMP vaccine administered via the nasal route or with parenteral priming followed with nasal boosting induced strong systemic and mucosal IgG and IgA responses and Th1, Th17 and cytotoxic T cells in the draining and peripheral LN.

**Fig. 2:**
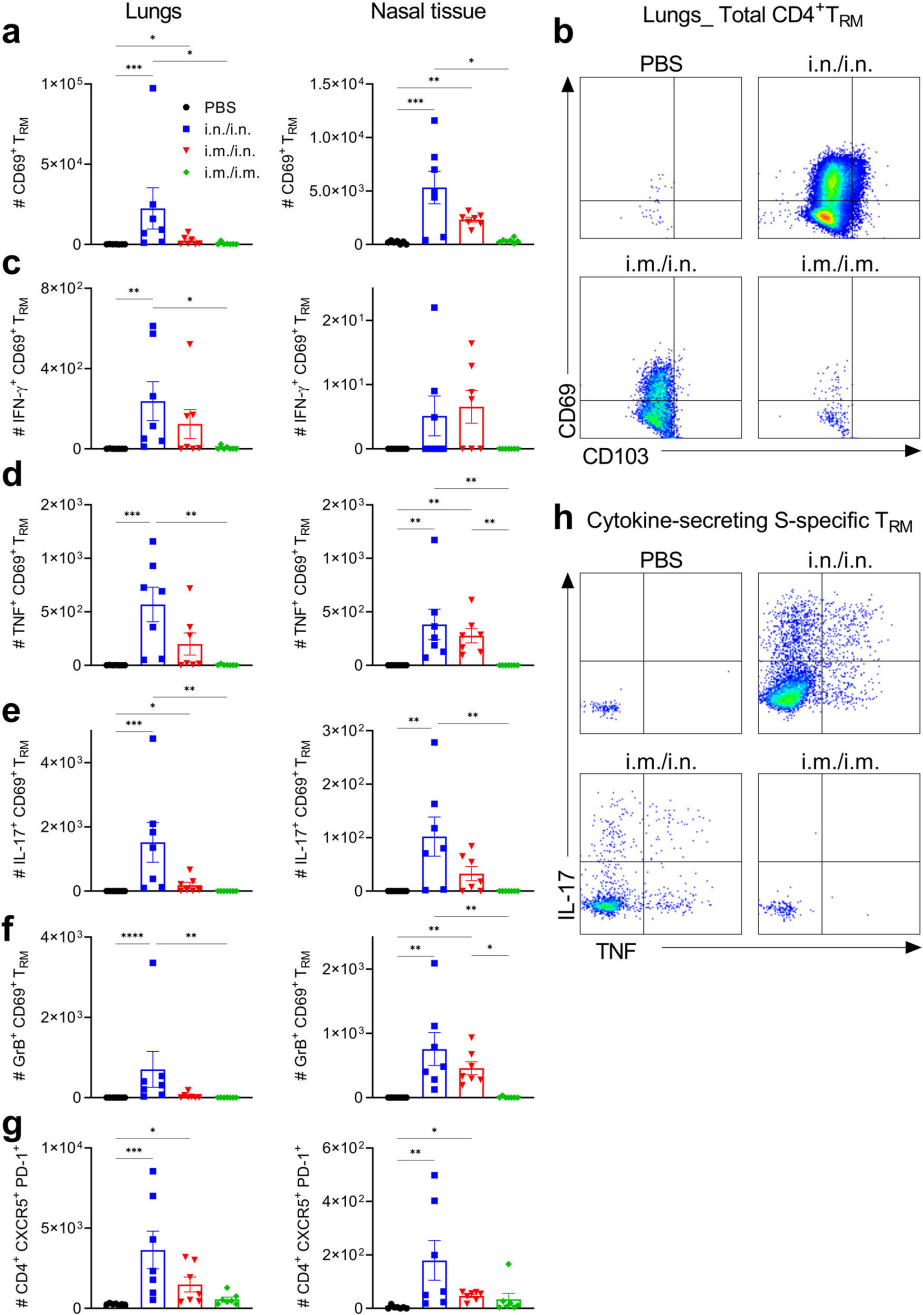
Immunization with Spike-LP-GMP by i.n. route elicits CD4^+^ T_RM_ and Tfh cells in the respiratory mucosa. Mice were immunized as described in Fig. 1, lung and nasal tissue samples were collected on day 35. **a** Number of CD4^+^ T_RM_ cells (CD44^+^CD62L^-^CD45^-^CD69^+^) in the lung and nasal tissue. **b** Representative flow cytometry plot of lung CD4^+^ T_RM_ cells. **c-f** Number of Spike-specific CD4^+^ T_RM_ cells secreting IFN-γ **c**, TNF **d**, IL-17 **e**, or GrB **f** in the lung and nasal tissue following 18 h stimulation with Spike trimer (2.5 µg/ml). **g** Representative flow cytometry plot of IL-17 and TNF secreting lung CD4^+^ T_RM_ cells. **h** Number of CD4^+^ Tfh cells in the lung and nasal tissue. Data were analyzed by one-way ANOVAs followed by post hoc Tukey’s test for multiple comparisons (*P < 0.05, **P < 0.01, ***P < 0.001, and ****P < 0.0001), error bars, SEM. Results are representative of three independent experiments.

**Fig. 3:**
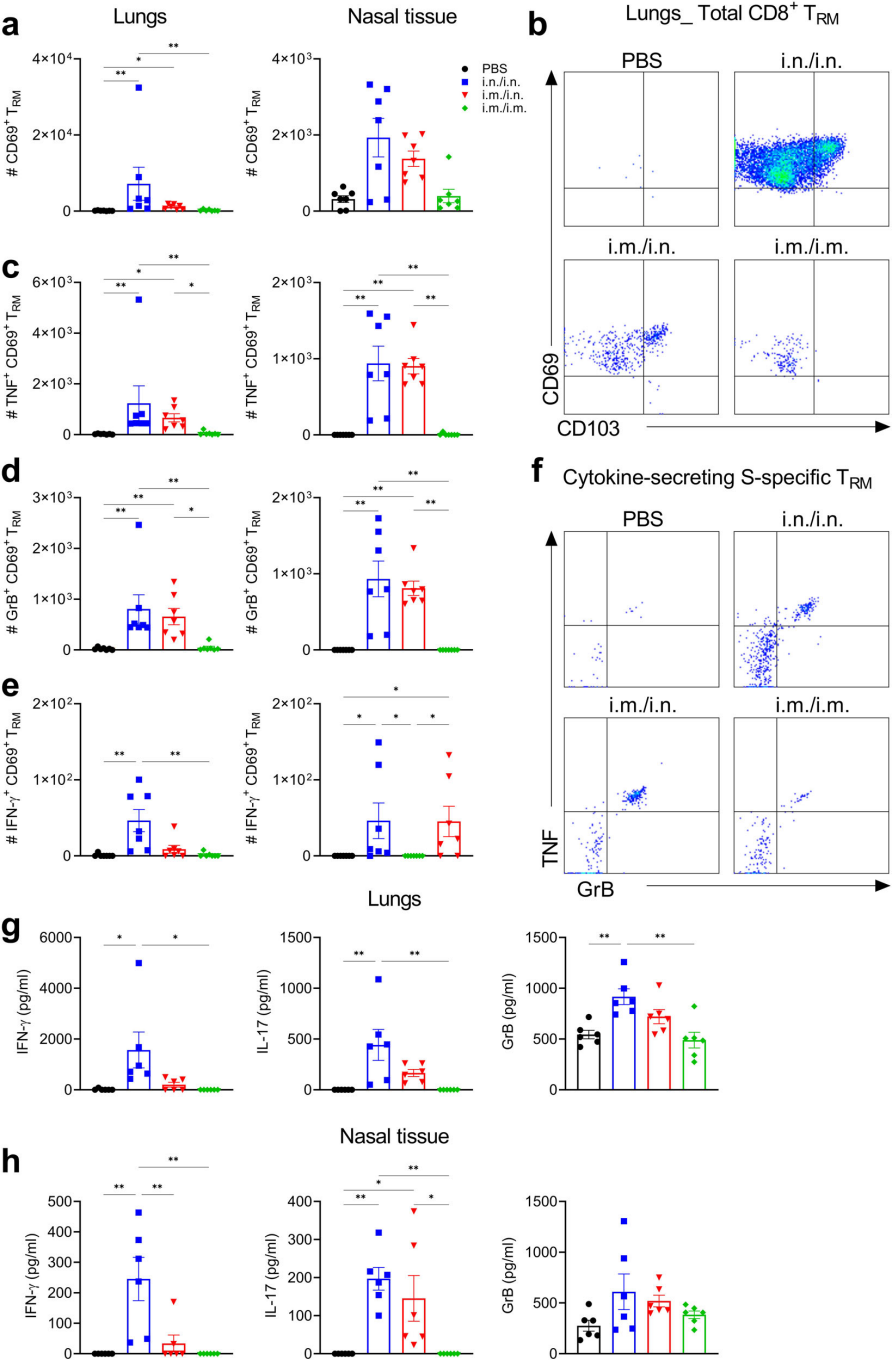
Immunization with Spike-LP-GMP by i.n. route elicits CD8^+^ T_RM_ in the respiratory mucosa. Mice were immunized as described in Fig. 1, lung and nasal tissue samples were collected on day 35 and stimulated for 18 h with Spike trimer (2.5 µg/ml). Supernatants were collected for cytokine quantification by ELISA and cells analyzed by flow cytometry **a** Number of CD8^+^ T_RM_ cells (CD44^+^CD62L^-^CD45^-^CD69^+^) in the lung and nasal tissue. **b** Representative flow cytometry plot of lung CD8^+^ T_RM_ cells. **c**–**e** Number of Spike-specific CD8^+^ T_RM_ cells secreting TNF **c**, GrB **d**, or IFN-γ **e** in the lung and nasal tissue. **f** Representative flow cytometry plot of TNF and GrB secreting CD8^+^ T_RM_ cells (lung). Antigen-specific IFN-γ, IL-17 and GrB production quantified by ELISA on supernatants of lung **g** and nasal tissue **h** cells stimulated for 18 h with Spike trimer (2.5 µg/ml). Data were analyzed by one-way ANOVAs followed by post hoc Tukey’s test for multiple comparisons (*P < 0.05, **P < 0.01, ***P < 0.001, and ****P < 0.0001), error bars, SEM. Results are representative of three independent experiments.

Accumulation of T_RM_ cells in the respiratory mucosa correlates with protection against respiratory pathogens^28^. Furthermore, CD4^+^ T_RM_ cells persist in the lungs of humans for up to 10 months after infection with SARS-CoV-2^18^. Analysis of T cell responses in the respiratory tract using flow cytometry (Gating strategy shown in Supplementary Fig. 3) revealed that CD4^+^ T_RM_ and CD8^+^ T_RM_ accumulated in the lung and nasal mucosa following intranasal immunization or heterologous prime-boost but not following parenteral immunization of mice with Spike-LP-GMP (Figs. 2a, b and 3a, b). To determine the specificity of the CD4^+^ T_RM_ and CD8^+^ T_RM_ generated, we restimulated immune cells from the lungs and nasal tissue of immunized mice with Spike trimer and performed intracellular staining for cytokines and granzyme B (GrB). We found that Spike-specific CD4^+^ T_RM_ cells were induced following two i.n. immunizations and produced IFN-γ, IL-17, TNF and GrB (Fig. 2c–g and supplementary Fig. 4a). Spike-specific CD8^+^ T_RM_ cells secreting TNF, GrB and IFN-γ were also induced following antigen restimulation (Fig. 3c–f and supplementary Fig. 4b). Responses were weaker after i.m. priming and i.n. boosting and were almost undetectable in mice given two immunizations by the i.m. route. Cytokine concentrations were also quantified by ELISA after stimulation of lung and nasal tissue cells with antigen for 18 h. The results demonstrate that i.n./i.n. and to a lesser extent i.m./i.n. immunization with Spike-LP-GMP induced significant Spike-specific IFN-γ, IL-17 and GrB production (Fig. 3g, h).

We also assessed induction of CD4^+^ T follicular helper (Tfh) cells, which play a key role in the development and maintenance of antibody-secreting B cells^29,30^. We found that the Spike-LP-GMP vaccine induced CD4^+^ Tfh cells (CD4^+^ CXCR5^+^ PD-1^+^) following i.n./i.n. and to a lesser extent following i.m./i.n., but not following i.m./i.m., immunization (Fig. 2h). IL-21-secreting tissue-resident CD4^+^ T cells were also found in the lungs and nasal tissue of mice immunized i.n./i.n. or i.m./i.n. with Spike-LP-GMP (Supplementary Fig. 5a–e). These findings demonstrate that delivery of our candidate vaccine by the nasal route or using a heterologous prime-boost strategy is effective for inducing CD4^+^ T_RM_ cells and Tfh cells in the respiratory tract.

### The Spike-LP-GMP vaccine induced long-lasting systemic and mucosal immune responses in mice

**Fig. 4:**
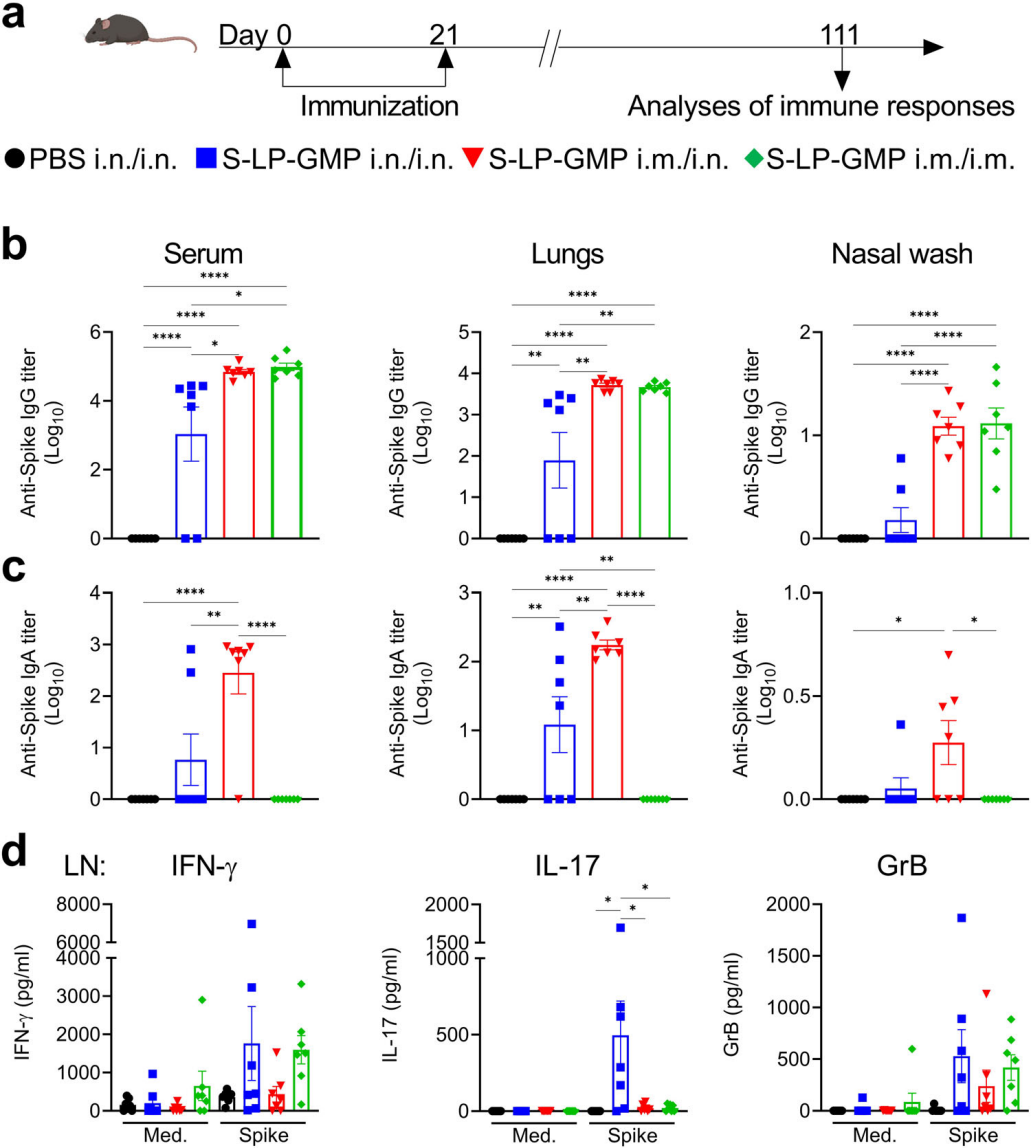
The Spike-LP-GMP vaccine induces persistent systemic and mucosal immune response in mice. **a** Schematic of experimental design; C57BL/6 mice (*n* = 7/group) were immunized twice (0 and 21 days) i.n./i.n., i.m./i.n. or i.m./i.m. with the Spike-LP-GMP vaccine or PBS and euthanized on day 111 for samples collection. Spike-specific IgG **b**, IgA **c** in the serum, lung homogenate and nasal wash. **d** Antigen-specific IFN-γ, IL-17 and GrB production by cervical and inguinal LN cells stimulated for 72 h with medium alone or Spike trimer (2.5 µg), quantified by ELISA. Data were analyzed by one-way ANOVAs followed by post hoc Tukey’s test for multiple comparisons (*P < 0.05, **P < 0.01, ***P < 0.001, and ****P < 0.0001), error bars, SEM. Results are representative of three independent experiments.

To evaluate the persistence of the immune responses induced by nasal immunization, mice were primed and boosted i.n. or primed i.m. and boosted i.n. with Spike-LP-GMP and immune responses were assessed 3 months later (Fig. 4a). The results indicated that Spike-specific IgG and IgA persisted in the serum and lung at least 3 months following immunization with Spike-LP-GMP via the i.n./i.n., i.m./i.n. and i.m./i.m. routes (Fig. 4b, c). However, the antibody responses were strongest in mice immunized using the heterologous prime-boost and parenteral approaches. Significant levels of Spike-specific IgA were only detected in the nasal wash of mice primed i.m. and boosted i.n.

**Fig. 5:**
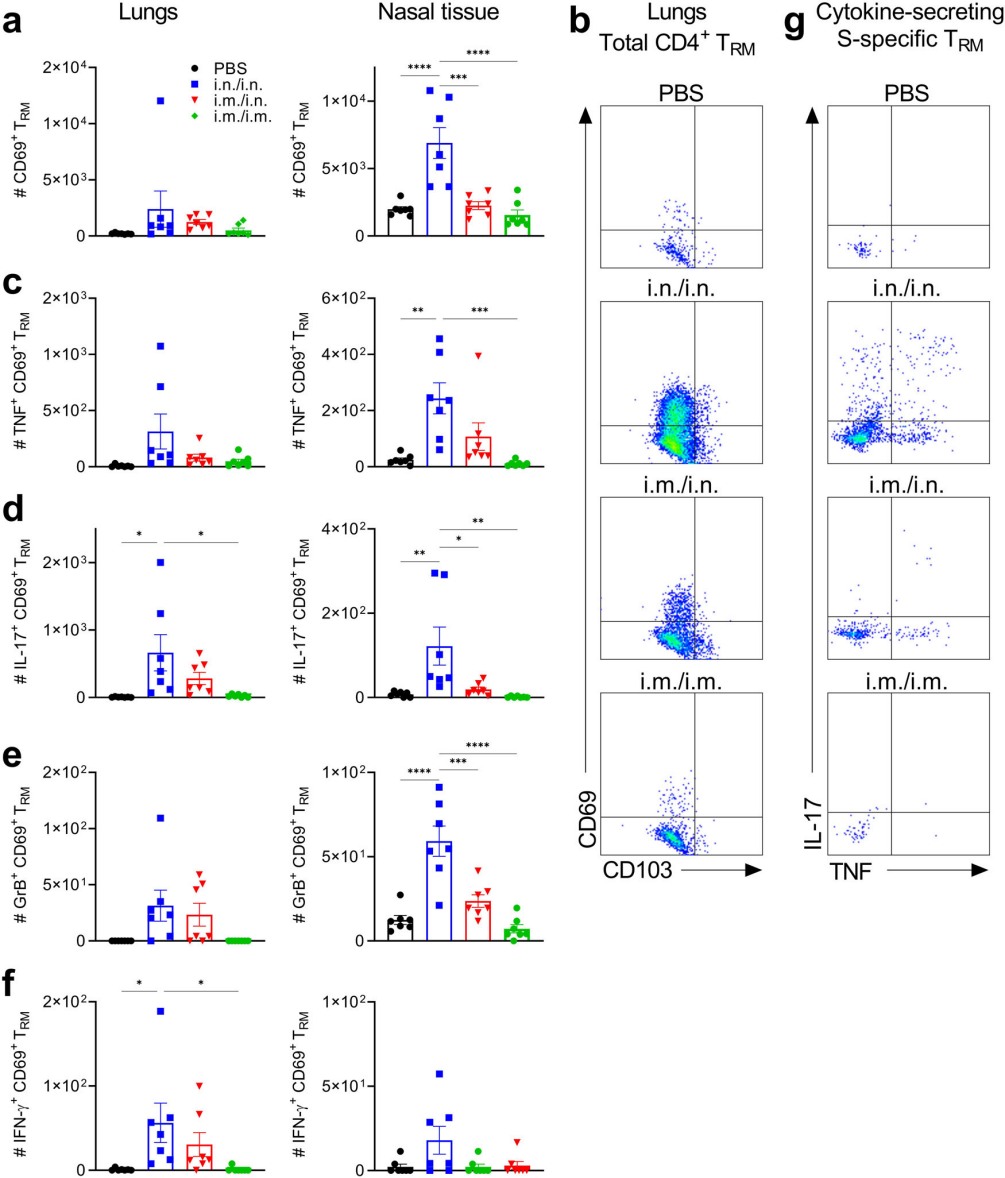
Spike-specific T_RM_ cells persist in the lungs and nasal tissue for at least 3 months post immunization. Mice were immunized as described in Fig. 4, lung and nasal tissue samples were collected on day 111. **a** Number of CD4^+^ T_RM_ cells (CD44^+^CD62L^-^CD45^-^CD69^+^) in the lung and nasal tissue. **b** Representative flow cytometry plot of CD4^+^ T_RM_ cells from lung. Number of Spike-specific CD4^+^ T_RM_ cells secreting TNF **c**, IL-17 **d**, GrB **e**, or IFN-γ **f** in the lung and nasal tissue following 18 h stimulation with Spike trimer (2.5 µg/ml), **g** Representative flow cytometry plot of IL-17 and TNF secreting CD4^+^ T_RM_ cells from lung. Data were analyzed by one-way ANOVAs followed by post hoc Tukey’s test for multiple comparisons (*P < 0.05, **P < 0.01, ***P < 0.001, and ****P < 0.0001), error bars, SEM.

Assessment of peripheral T cells responses 3 months after immunization revealed that Spike-specific IFN-γ, IL-17 and GrB were still detectable in draining LN in immunized mice, but antigen-specific cytokine production was strongest in mice primed and boosted by the i.n. route (Fig. 4d). Quantification of CD4^+^ T_RM_ and CD8^+^ T_RM_ in respiratory tissue revealed a significant population of CD4^+^ T_RM_ and CD8^+^ T_RM_ in the nasal tissue and smaller numbers in the lungs after i.n./i.n. immunization (Fig. 5a, b and Supplementary Fig. 6a). These CD4^+^ T_RM_ cells produced TNF, IL-17, GrB and IFN-γ following in vitro restimulation with Spike trimer antigen (Fig. 5c–g). The CD8^+^ T_RM_ cells produced GrB and TNF following stimulation with Spike trimer (Supplementary Fig. 6a-d). Overall, our findings demonstrate that Spike-specific immune responses persist in the respiratory tissue for up to 3 months after intranasal immunization with Spike-LP-GMP.

### The Spike-LP-GMP vaccine protected K18-hACE2 transgenic mice against lethal infection following challenge with ancestral or Delta strains of SARS-CoV-2

**Fig. 6:**
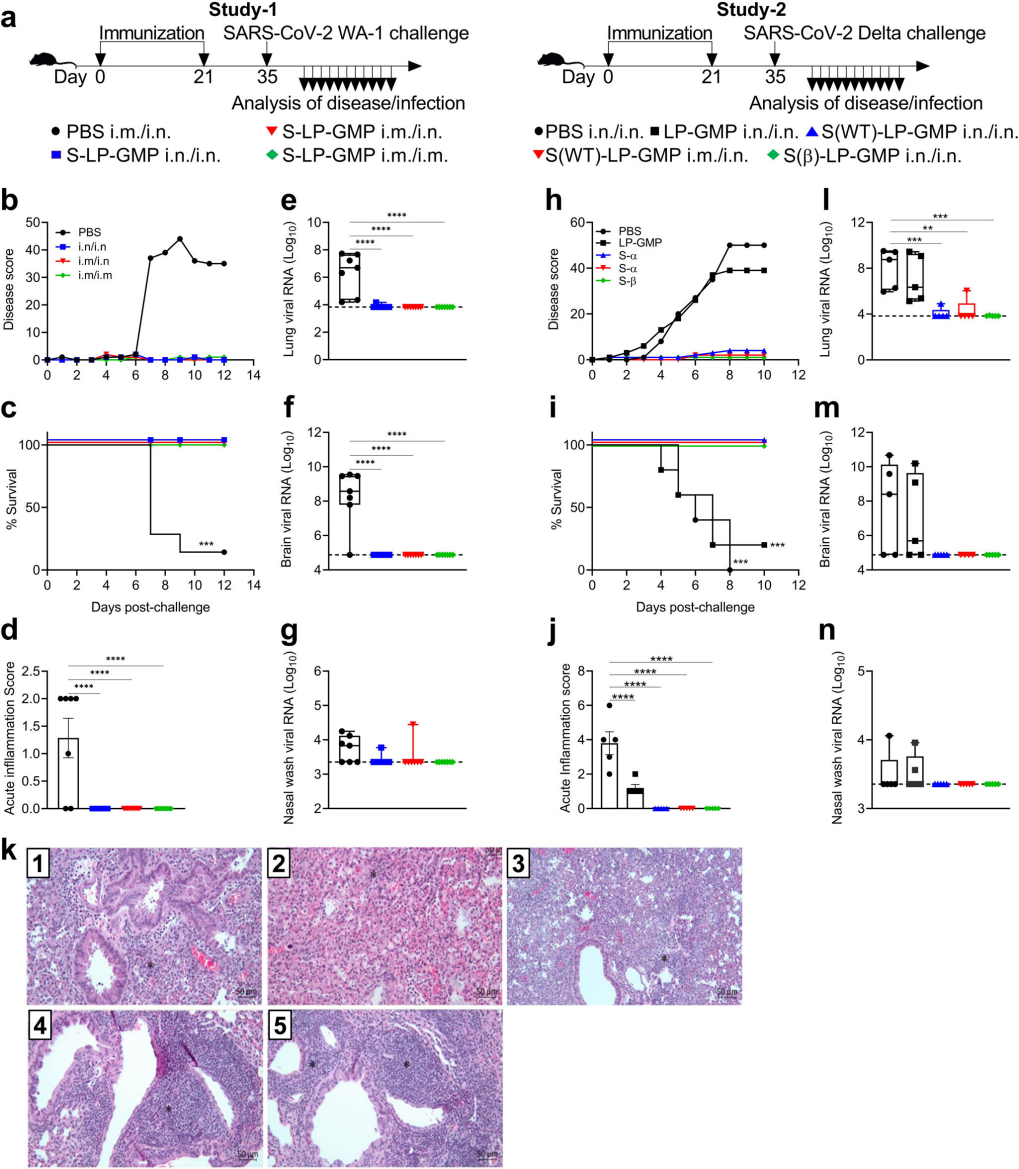
The Spike-LP-GMP vaccine protects K18-hACE2 transgenic mice against challenge with SARS-CoV-2 variants. **a** Experimental design. In study 1, K18-hACE2 (*n* = 8/group) were immunized twice (0 and 21 days) i.n./i.n., i.m./i.n. or i.m./i.m. with the Spike-LP-GMP vaccine (WT virus strain) or PBS and challenged with SARS-CoV-2 WA-1 on day 35. Disease score (based on weight loss, appearance, activity, eye closure, respiration, and hypothermia) **b** and survival **c** and lung acute inflammatory score **d**. Virus RNA copies in the lung **e** brain **f** and nasal wash **g** 12 days after SARS-CoV-2 WA-1 challenge. Dashed line indicates limit of detection by qPCR. In study 2, K18-hACE2 mice were immunized twice (0 and 21 days) i.n./i.n. or i.m./i.n. with the Spike-LP-GMP vaccine (WT or Beta virus strain) or PBS and challenged with SARS-CoV-2 Delta on day 35. Disease score **h** and survival **i** and lung acute inflammatory score **j**, with representative images of H&E stained lung sections (PBS (1), LP-GMP (2), Spike(WT)-LP-GMP i.m./i.n. (3), Spike(WT)-LP-GMP i.n./i.n. (4) and Spike(β)-LP-GMP i.n./i.n. (5) **k**. Virus RNA copies in the lung **l**, brain **m**, and nasal wash **n** 10 days after SARS-CoV-2 Delta challenge. Dashed line indicates limit of detection by qPCR. Data were analyzed by one-way ANOVAs followed by post hoc Tukey’s test for multiple comparisons (*P < 0.05, **P < 0.01, ***P < 0.001, and ****P < 0.0001), error bars, SEM. Log-rank Mantel-Cox test was used to determine statistical significance for survival analyzes (***P < 0.001).

We next assessed the ability of Spike-LP-GMP vaccine to protect against lethal challenge with SARS-CoV-2 WA-1 or delta strains in K18-hACE transgenic mice that express human ACE-2, which develop lethal COVID-19-like disease^25^. In the first study, K18-hACE2 mice were immunized i.n./i.n., i.m./i.n. or i.m./i.m. with Spike-LP-GMP (Wuhan/wild-type (WT) strain) on days 0 and 21 and challenged i.n. 14 days later with 10^4^ PFU/mouse SARS-CoV-2 WA-1 (Fig. 6a). Control mice immunized with PBS and challenged with SARS-CoV-2 WA-1 rapidly developed severe disease and 9 of 10 mice succumbed to infection by day 12 post-challenge (Fig. 6b, c). In contrast, the Spike-LP-GMP immunized mice did not show signs of disease or distress; their disease scores evaluated using the method described by Wong et al.^25^, were at baseline throughout the study and all mice survived to the end of the experiment (Fig. 6b, c). Histological analyses of lung sections following SARS-CoV-2 challenge revealed significant acute inflammation in mice immunized with PBS and challenged with SARS-CoV-2 WA-1(Fig. 6d). In contrast, mice immunized with the Spike-LP-GMP vaccine by i.n./i.n., i.m./i.n. and i.m./i.m. routes had low overall inflammation scores following challenge with the SARS-CoV-2 WA-1.

We assessed the viral RNA burden in the lung, brain and nasal wash by quantification of RNA copies of SARS-CoV-2 nucleocapsid. High virus RNA copies were detected in the lung and brain of PBS immunized mice after challenge with SARS-CoV-2 WA-1, whereas viral RNA was at the limit of detection in the lung and brain of mice immunized with Spike-LP-GMP by i.n./i.n., i.m./i.n. and i.m./i.m. routes (Fig. 6e, f). Viral RNA in the nasal wash was also lower in Spike-LP-GMP immunized mice compared with PBS immunized mice, but these differences were not statistically significant (Fig. 6g).

In the second SARS-CoV-2 challenge study, we used the Delta variant (B.1.617.2), which is more virulent and lethal in humans and in K18-hACE2 mice and we also included an experimental vaccine with the Spike protein based on the SARS-CoV-2 Beta variant. Here mice were immunized with PBS i.n./i.n., LP-GMP alone i.n./i.n., Spike(WT)-LP-GMP i.m./i.n., Spike(WT)-LP-GMP i.n./i.n., Spike(β)-LP-GMP i.n./i.n. at days 0 and 21. Fourteen days later, the animals were intranasally challenged with 10^4^ PFU/dose SARS-CoV-2 Delta variant (Fig. 6a). Mice immunized with PBS or adjuvant only (LP-GMP) rapidly developed disease following viral challenge and 7 of 8 mice immunized with PBS were morbid/moribund and required humane euthanasia by day 8 (Fig. 6h, i). In contrast, SARS-CoV-2 Delta challenge of mice immunized i.n./i.n. or i.m./i.n. with Spike-LP-GMP did not show any signs of disease over the duration of the experiment and 100% of mice survived. Histological analysis revealed severe acute inflammation in the lung in PBS-immunized control mice following challenge with the SARS-CoV-2 Delta variant (Fig. 6j, k). Mice immunized with LP-GMP had mild acute inflammation in the lung following challenge with the SARS-CoV-2 Delta variant. In contrast, acute inflammation was undetectable after SARS-CoV-2 challenge of mice immunized with Spike(WT)-LP-GMP i.m./i.n., Spike(WT)-LP-GMP i.n./i.n., Spike(β)-LP-GMP i.n./i.n. (Fig. 6j, k).

Assessment of RNA copies of SARS-CoV-2 nucleocapsid revealed that immunization with the experimental vaccine based on WT or Beta strains by nasal or heterologous prime-boost strategies significantly reduced the viral RNA copies in the lungs compared with control mice after challenge with Delta strain (Fig. 6l, m). The viral RNA copy numbers were also reduced to the limit of detection in brain and nasal wash of mice immunized i.n./i.n., i.m./i.n. or i.m./i.m. with the Spike-LP-GMP vaccine (Fig. 6n). These findings demonstrate that immunization with the Spike-LP-GMP vaccine delivered by the i.n. route or using heterologous prime-boost strategy is highly effective at preventing disease, and dissemination of virus into the lung and brain.

### Immunization with Spike-LP-GMP induced a breadth of cross-neutralizing antibodies against SARS-CoV-2 VOC

**Fig. 7:**
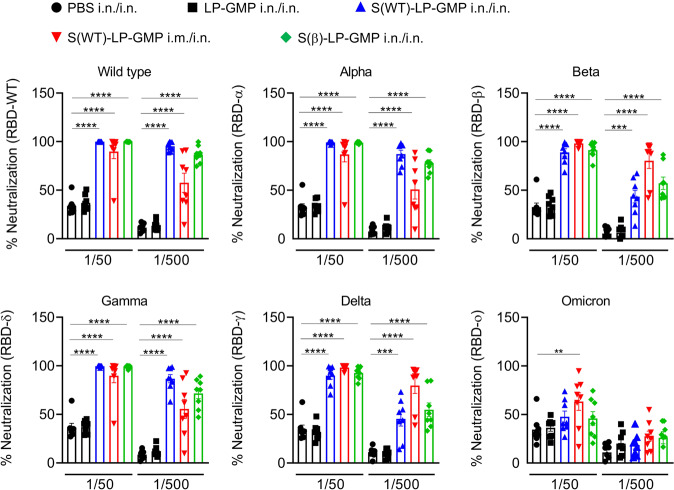
Antibodies induced by nasal Spike-LP-GMP vaccine neutralize multiple variants of SARS-CoV-2 but have reduced efficacy against Omicron. Serum neutralizing antibody responses, based on inhibition of binding of SARS-CoV-2 RBD Spike from WT, Alpha, Beta, Delta, Gamma and Omicron to hACE-2 (MSD assay) were assessed on day 35 (before SARS-CoV-2 challenge), 2 weeks after i.n./i.n. or i.m./i.n. immunization with the Spike-LP-GMP vaccine (WT or Beta virus strains) or PBS. Data are represented as percentage of neutralization. Data were analyzed by one-way ANOVAs followed by post hoc Tukey’s test for multiple comparisons (*P < 0.05, **P < 0.01, ***P < 0.001, and ****P < 0.0001), error bars, SEM.

We assessed the capacity of the antibodies in mice immunized with our vaccine to inhibit binding of SARS-CoV-2 RBD Spike (from different VOC) to human ACE-2 as a surrogate of virus neutralization. Serum from mice immunized with the Spike(WT)-LP-GMP i.n./i.n., Spike(β)-LP-GMP i.n./i.n. or Spike(WT)-LP-GMP i.m./i.n. vaccines and challenged with SARS-CoV-2 Delta had significant virus neutralizing activity against WT and Alpha, Beta, Gamma and Delta variants of SARS-CoV-2 (Fig. 7). However, the ability to neutralize Omicron was dramatically reduced. These findings demonstrate that antibodies induced by i.n. immunization with the Spike-LP-GMP are highly effective at neutralizing most SARS-CoV-2 VOC but are less effective against Omicron.

## Discussion

This study has demonstrated that i.n. immunization or parenteral priming following i.n. boosting with an experimental COVID-19 vaccine, based on the SARS-CoV-2 Spike trimer and formulated with a novel adjuvant, is an effective approach for inducing local antibody and T cell responses in the respiratory tract. Furthermore, immunization with the Spike-LP-GMP vaccine by i.n./i.n. or i.n./i.m. or i.m./i.m routed prevented lethal infection with the ancestral strain and Delta variant of SARS-CoV-2. However, only the i.n./i.n. or i.n./i.m. protocols induce mucosal IgA and respiratory TRM cells and may therefore, be more effective at inducing sustained sterilizing immunity in the respiratory mucosa.

While vaccines have significantly reduced the incidence of many infectious diseases and eliminated smallpox, some fail to prevent infection with the target pathogen and allow community transmission. For example, current acellular pertussis vaccines prevent whooping cough but do not prevent nasal colonization with *B. pertussis*^21,31^. There are several possible explanations for this, but a prominent one is a failure of current parenterally delivered pertussis vaccines to induce mucosal immunity, including induction of IgA and T_RM_ cells in the respiratory tract, which provide the first line of defense against re-infection of mucosal surfaces. Genetic mutations or deletions in protective antigens also reduce effectiveness of antibodies and T cell responses. The effects of immune-driven selective pressure have been well established for influenza virus, where mutations in the hemagglutinin protein can dramatically reduce neutralizing antibodies and CD4^+^ T cell responses specific for this protective antigen^32^. Current licensed mRNA and adenovirus-vectored COVID-19 vaccines prevent severe COVID-19 disease and hospital admissions, but do not prevent respiratory infection or transmission of SARS-CoV-2. This has mainly been attributed to escape from vaccine-induced immunity through the emergence of SARS-CoV-2 variants that have a significant number of mutations in the Spike protein, especially in the RBD receptor binding domain, a major target for virus neutralizing antibodies. However, the phase 3 efficacy trials conducted at the time when the virus had not significantly mutated showed that several COVID-19 vaccines generated high levels of protection against COVID-19 disease, but in most cases did not assess prevention of SARS-CoV-2 infection in the respiratory tract^6^. Studies in non-human primates, where SARS-CoV-2 challenge was performed at the peak of the immune response with a virus homologous to the vaccine, revealed that many of the COVID-19 vaccines could prevent lung infection, but failed to induce sterilizing immunity in the nasal cavity^33–36^. Furthermore, breakthrough infections are seen in vaccinated humans, leading to acute respiratory failure in frail patients^37^. All current licensed COVID-19 vaccines are administered parenterally.

Nasal delivery of vaccines, such as the licensed nasal spray influenza vaccine FluMist, has several advantages over parenteral delivery, including ease of administration, and importantly mimics natural infection, inducing mucosal immunity in the upper respiratory tract, including secretory IgA^38^. Mucosal IgA induction following vaccination seems to be crucial in promoting sterilizing immunity against SARS-CoV-2^39^. Nasal IgA wanes quickly following severe COVID-19 and is not boosted by subsequent immunization with COVID-19 adenoviral vector or mRNA vaccines^40^, suggesting that parenteral COVID-19 vaccines fail to induce or boost pre-existing local mucosal immunity. Mucosal immunization also has the advantage of inducing mucosal IgA at distal mucosal sites, which in the case of SARS-CoV-2 infection, may help to alleviate its negative impact on the gastrointestinal tract^41^. Serum neutralizing antibodies induced by parenteral immunization need to transudate across the respiratory mucosa to prevent infection with SARS-CoV-2, whereas local IgG and IgA induced by respiratory infection or nasal vaccine may provide a more efficient first line of defense against respiratory pathogens. Nasal vaccines also have the capacity to induce CD4^+^ and CD8^+^ T_RM_ cells in the respiratory tissues. Lung CD4^+^ T_RM_ cells confer long-term immunity and protect mice against a lethal infection with influenza virus^42^. Our study demonstrates that two doses of Spike-LP-GMP delivered by the i.n. route, or with a single i.n. booster following parenteral priming, induces systemic and local IgG and IgA antibodies and nasal and lung CD4^+^ and CD8^+^ T_RM_ cells, including Tfh cells, required for B cell production of antibodies. Furthermore, the nasal vaccine conferred complete protection against COVID-19-like disease and infection of the lungs and brain, and substantially reduced the viral load in the nasal wash.

One of the disadvantages of protein subunit vaccines is that they are poorly immunogenic and can induce tolerance when delivered by mucosal routes^43,44^. This can be overcome by the addition of adjuvants and/or delivery systems that retain the antigen at the site of immunization, promote phagocytic uptake by antigen-presenting cells and stimulate innate immune responses that direct induction of adaptive immunity. Although alum is the classic adjuvant for injectable vaccines, especially those against bacterial infections, it does not effectively promote T cell responses. Other adjuvants, such as MF59, Alum in combination with CpG 1018 (used in Corbevax and SCB-2019 COVID-19 vaccines) and Matrix-M (used in the Novavax COVID-19 vaccine), are effective at boosting T cells and antibody responses to parenterally delivered protein subunit vaccines^45–49^.The current study, as well as our published reports on a pertussis vaccine^20^, demonstrate that LP-GMP is a potent adjuvant for protein vaccines delivered by parenteral or nasal routes. Other studies show that i.n. administration of a synthetic DNA plasmid expressing Spike trimer with a chemokine adjuvant is effective at inducing mucosal antibodies and T cells^50^. A liposomal formulation containing TLR4 and TLR7 agonists was also shown to be an effective mucosal adjuvant for an influenza vaccine in mice^51^.

Novel adjuvants in nasal vaccines for human use will require stringent assessment of safety. Adverse events have been reported following i.n. immunization of humans with an influenza virus vaccine formulated with the bacterial toxin LTK63 as an adjuvant^27^. However, it has been attributed to the specific bacterial toxin rather than the route of immunization. It has been demonstrated that COVID-19 mRNA vaccines can induce mucosal immune responses following parenteral priming and nasal boosting^52^. However, the nasal mucosal environment of humans could pose an issue for the efficacy for a non-adjuvanted protein vaccine in naive individuals. An intranasally delivered adenovirus-vectored COVID-19 vaccine had low immunogenicity and failed to protect against SARS-CoV-2 infection in a phase I clinical trial^53^. Our experimental Spike LP-GMP subunit vaccine can overcome the low immunogenicity of antigens delivered by the nasal route. Mucosal vaccines have been safely used for some decades in veterinary practices^54,55^. The nasal influenza vaccine FluMist is safe and effective in humans^56^. Our data also shows that nasal immunization if mice with the Spike-LP-GMP experimental vaccine did not induce significant concentrations of inflammatory cytokines in the respiratory mucosa or serum. The rapid waning of vaccine-induced protective immunity against symptomatic and asymptomatic infection with SARS-CoV-2 reflects the rapid decline in serum neutralizing antibody responses within months of vaccination. This has necessitated the implementation of booster vaccination to enhance circulating virus antibody responses. While mutations in the Spike protein have reduced the effectiveness of vaccines over time, it also appears that current parenterally delivered COVID-19 vaccines are not effective at promoting immunological memory, especially in the respiratory tract. The present study along with previous reports on vaccines against SARS-CoV-2 and other pathogens, have demonstrated that nasal delivery can induce T_RM_ cells in the lungs. In addition, mucosal resident T cells were found only in people experiencing breakthrough infection but not in non-infected vaccinated individuals^57^. Our findings extend these observations to show that a candidate nasal COVID-19 vaccine can also induce T_RM_ cells in the nasal tissue. T_RM_ cells play a key role in maintaining long term immunity in the upper and lower respiratory tract. We found that the Spike-specific respiratory T_RM_ cells secrete IFN-γ, TNF, IL-17 and GrB and may therefore mediate direct anti-viral cellular immunity. Tfh cells were also induced in the lungs and nasal tissue and these cells may play a crucial role in kick-starting B cell production of local antibodies after circulating levels have declined post vaccination.

It has been reported that T cell epitopes are highly conserved between Wuhan and Omicron strains^58,59^. In contrast, many neutralizing antibody epitopes are in variable regions of the Spike trimer and recent virus variants, especially the Omicron subvariants BA-1 to BA.5, have mutations on the RBD that reduce the neutralizing capacity of antibodies generated against the ancestral sequences in the current vaccines^60^. Consistent with this, we found that antibodies induced with the ancestral (WT) sequence of the Spike protein in our experimental vaccine were less effective at neutralizing Omicron. This highlights the need to use more recent versions of the Spike trimer or to add other antigens, such as Nucleocapsid, ORF and other proteins that are less mutated than Spike, in future COVID-19 vaccine formulations^61^. Since virus variants escape antibodies more readily than T cells, a vaccine that induces T cells as well as antibodies, especially in the respiratory tract may have significant advantage. Collectively, our study suggests that nasal protein vaccine formulated with a potent adjuvant may be a useful approach to confer long-term protection against SARS-CoV-2 in humans.

## Methods

### Mice

C57BL/6 female mice (8 to 12 weeks old) were bred in-house. All animal experiments performed at Trinity College Dublin were conducted in accordance with European Union regulations and under license (AE19136/P130) from the Irish Health Products Regulatory Authority with approval from the Trinity College Dublin Bioresources Ethics Committee. SARS-CoV-2 challenge studies in B6.Cg-Tg(K18-ACE-2)2Prlmn/J mice were conducted in the West Virginia University Biosafety Laboratory Level 3 facility under the IBC protocol number 20-09-03/IACUC protocol number 2009036460.

### Vaccine formulation

The experimental vaccine Spike-LP-GMP consists of the full-length trimeric SARS-CoV-2 Spike protein (3 µg/mouse) corresponding to the original Wuhan-1 or the Beta strain, manufactured by Peak proteins (United Kingdom) and LP-GMP as the adjuvant. It includes the STING agonist C-di-GMP (10 µg/mouse; Invivogen, France), and the TLR2 agonist LP1569 (50 µg/mouse; manufactured by EMC Microcollections, Germany).

### Immunization protocol and samples collection

Mice were immunized twice, 21 days apart, with Spike-LP-GMP (3 µg of Spike trimer, 10 µg of C-di-GMP, 50 µg of LP1569) by i.m./i.m., i.n./i.n or i.m./i.n. routes For i.n immunization, mice were anesthetized with ketamine and xylazine prior to immunization. The i.m. vaccine was administered in 50 µl to the quadricep muscles and i.n. vaccine was administered in 24 or 50 µl. Mice were euthanized 14 or 90 days after the last immunization. Serum, lung (left and post-caval lobes) and nasal wash were collected for analysis of antibody responses. Nasal tissue, 3 right lung lobes, cervical and inguinal LN, and spleen were collected for analysis of T cell responses.

### Flow cytometry analysis on lung and nasal tissue T cells

Mice were injected i.v. with 0.75 µg of anti-CD45-PE (Biolegend) 10 min before euthanasia to differentiate between tissue-resident and circulating immune cells. Lung and nasal tissue were digested for 1 h with DNase I (20 U/mL Sigma-Aldrich) and collagenase-D (1 mg/mL, Roche) at 37 °C under agitation. The degraded tissues were forced through a 40 µm cell strainer to generate a single-cell suspension. Red blood cells were lysed using ACK buffer, centrifuged and resuspended in complete RPMI medium [RPMI 1640 (Gibco) supplemented with 10% FBS (GE Healthcare Life Sciences, Logan, UT), 1 mM sodium pyruvate, 50 µM β-mercaptoethanol, 1% streptomycin/penicillin (all Gibco)]. One-half of each cell suspension was used for flow cytometry staining with antibodies (Supplementary Table 1) for 20 min at room temperature. Cells were fixed and permeabilized (eBioscience™ Intracellular Fixation & Permeabilization Buffer Set, Thermo Fisher Scientific) according to the manufacturer’s protocol and stained with intracellular antibodies for 30 min at room temperature. After washing, the cells were analyzed by spectral flow cytometry. The other half of the single-cell suspension was restimulated with 2.5 µg/ml of Spike protein, anti-CD28 and anti-CD49d (1 µg/ml) (Thermo Fisher Scientific) or antibodies only for 18 h at 37 °C 5% CO_2_. Brefeldin A (5 µg/mL; Sigma-Aldrich) was added for the final 4 h of incubation. Cells were centrifuged and the supernatants were collected for cytokines analyzes and the cells were stained with surface antibodies (Supplementary Table 1) for 20 min at room temperature. Cells were fixed and permeabilized (eBioscience™ Intracellular Fixation & Permeabilization Buffer Set, Thermo Fisher Scientific) according to the manufacturer’s protocol before intracellular cytokines staining. After washing, samples were acquired on an Aurora spectral flow cytometer using SpectroFlo software (Cytek) and analyzed using Kaluza software (Beckman Coulter).

### Ex vivo antigen stimulation of draining lymph nodes cells

Single-cell suspensions from cervical and inguinal LN combined or alone (8 × 10^5^ cells/well) and spleen cells (from naive mice as a source of additional antigen presenting cells; 8 × 10^4^ cells/well) were cultured for 72 h at 37 °C in complete RPMI medium with Spike protein (2.5 µg/ml) or medium. Supernatants were collected and IFN-γ, IL-17 and GrB concentrations in supernatants were measured by ELISA according to the manufacturer’s instructions (R&D Systems or BD).

### Spike-specific antibodies by ELISA

Spike-specific IgG, IgA or IgG2c antibodies in serum, lung tissue or nasal tissue was determined by ELISA as described for pertussis antigens^20^, using plates coated with Spike trimer (1 µg/ml).

### Spike-ACE-2 binding inhibition assay

Antibodies in serum, lung and nasal wash of immunized mice were assessed for inhibition of binding of SARS-CoV-2 Spike to human ACE-2 as a surrogate of virus neutralization. The protocol was modified from that reported by Phelan et al.^62^. High binding ELISA plates (Cruinn) were coated with 50 µL per well of Spike trimer (1 µg/ml) for 2 h at room temperature. Plates were washed 3 times with PBS 0.5% Tween buffer. Blocking buffer (PBS 1% BSA; 50 µl/well) was added for 2 h at room temperature. Plates were washed 3 times with PBS 0.5% Tween buffer and dried on paper. Samples were incubated for 1 h and then biotinylated ACE-2 (Sino Biological, China) was added to each well except the blank well for an additional 1 h. Plates were washed 3 times with PBS 0.5% Tween buffer and dried on paper. 50 ml of streptavidin-HRP was added per well for 20 min at RT. Plates were washed 3 times with PBS 0.5% Tween buffer and dried on paper. 50 ml of TMB substrate solution (Thermo Fisher) was added per well. Plates were incubated in the dark until development. 25 µl of H_2_SO_4_ (stop solution) was added per well. Absorbance was measured at 450 nm on a Versamax microplate reader (Softmax Pro 7.0.3). Biotinylated ACE-2 alone was used as positive control (100% binding). Results were expressed as a percentage of neutralization, representing the inhibition of biotinylated ACE-2 binding by antibodies in samples.

### SARS-CoV-2 challenge

B6.Cg-Tg(K18-ACE-2)2Prlmn/J mice were immunized twice, 21 days apart, with Spike-LP-GMP (3 µg of Spike trimer, 10 µg of C-di-GMP, 50 µg of LP1569) in 50 µl by i.n./i.n or i.m./i.n. routes For i.n immunization, mice were anesthetized with ketamine and xylazine prior to immunization. Mice were challenged i.n. with SARS-CoV-2 in Laboratory Level 3 facility under the IBC protocol number 20-09-03. All mice were monitored daily and humanely euthanized based on the disease scoring system, monitoring weight loss, appearance, activity, eye closure, respiration, and hypothermia^25^. Challenged mice that were assigned a health score of 5 or above or reached the end of the experiment were euthanized with an i.p injection of Euthasol (Pentobarbital) followed by a secondary measure of euthanasia with cardiac puncture. Viral RNA burden in the lung, brain and nasal wash was determined by quantifying of RNA copies of SARS-CoV-2 nucleocapsid by qPCR using the Applied Biosystems TaqMan RNA to CT One Step Kit (Ref: 4392938). We utilized nucleocapsid primers (F: ATGCT

GCAATCGTGCTACAA; R: GACTGCCGCCTCTGCTC); and TaqMan probe (IDT:/56-FAM/TCAAGGAAC/ZEN/AACATTGCCAA/3IABkFQ/) as described^25^.

### Histopathology

Left lobes of lungs from each were fixed in 10 mL of 10% neutral buffered formalin. Fixed lungs were paraffin-embedded into 5 μm sections. Sections were stained with hematoxylin and eosin for histopathology analysis. Lung samples were scored for chronic and acute inflammation in the lung parenchyma, blood vessels, and airways. Acute inflammation was characterized by the presence of neutrophils and edema as previously described^25^.

### Meso scale discovery SARS-COV-2 ACE-2 neutralization assay

Serum from mice 2 weeks post-boost were analyzed using the SARS-CoV-2 Plate 7 Multi-Spot 96-well, 10 spot plate following the manufacturer protocol on the MSD QuickPlex SQ120. The 10 spots contained: (1) RBD Omicron (B1.1.529), (2) RBD Beta (B.1.351), (3) CoV-2 N, (4) RBD Gamma (P.1), (5) BSA, (6) RBD Alpha (B.1.1.7), (7) Spike P.1, (8) Spike B.1.1.7, (9) RBD Delta (B.1.617.2), and (10) CoV2 S1 RBD. Two dilutions of serum, 1:50, and 1:500, were analyzed on the MSD neutralization assay for each mouse to perform Area Under the Curve analysis on the electrochemiluminescence using GraphPad Prism. Results were expressed as a percentage of neutralization.

### Spike-specific secretory IgA antibodies by ELISA

C57BL/6 mice (*n* = 7/group) were immunized twice (0 and 21 days) i.n./i.n., i.m./i.n. or i.m./i.m. with the Spike-LP-GMP vaccine or PBS and euthanized on day 35. Lung, nasal wash and NALT (nasal associated lymphoid tissue, 72 h stimulation of NALT with 2.5 µg/ml of Spike trimer) were collected. Spike-specific secretory IgA antibodies in nasal wash, lung homogenate and NALT supernatant were determined by ELISA. Medium binding ELISA plates (Cruinn) were coated with Spike trimer (1 µg/ml) (Peak protein, United Kingdom) for 2 h at room temperature. Plates were then washed 3 times with PBS 0.5% Tween buffer and dried on paper. In total 50 ml of blocking buffer (PBS + 1% BSA) per well was added for 2 h at room temperature. Plates were washed 3 times with PBS 0.5% Tween buffer and dried on paper. Samples were incubated for 2 h at room temperature. Plates were then washed 3 times with PBS 0.5% Tween buffer and dried on paper. A total of 50 µl of anti-mouse secretory component (IgA) antibody coupled with HRP (Assay Genie Ref. MOES01461) was added for 1 h at room temperature. Plates were washed 3 times with PBS 0.5% Tween buffer and dried on paper. A total of 50 µl of TMB substrate solution (Thermo Fisher) was added per well. Plates were incubated at room temperature in the dark until development. A total of 25 µl of H_2_SO_4_ (stop solution) was added per well. Absorbance was measured at 450 nm on a Versamax microplate reader (Softmax Pro 7.0.3).

### Statistical analysis

All statistical analyzes were performed using Graph-Pad Prism 9.0 Software. Data were presented as mean ± SEM. Data were analyzed by one-way ANOVAs followed by post hoc Tukey’s test for multiple comparisons. Log-rank Mantel-Cox test was used to determine statistical significance for survival analyzes. *P* values < 0.05 were considered significant.

### Reporting summary

Further information on research design is available in the Nature Research Reporting Summary linked to this article.

## Supplementary information

Supplementary Material

Reporting Summary

## Data Availability

The datasets generated during the current study are available from the corresponding author on reasonable request.

## References

[1] Baden LR (2021). Efficacy and safety of the mRNA-1273 SARS-CoV-2 vaccine. N. Engl. J. Med..

[2] Sadoff J (2021). Safety and efficacy of single-dose Ad26.COV2.S vaccine against Covid-19. N. Engl. J. Med..

[3] Shinde V (2021). Efficacy of NVX-CoV2373 Covid-19 Vaccine against the B.1.351 Variant. N. Engl. J. Med..

[4] Voysey M (2021). Safety and efficacy of the ChAdOx1 nCoV-19 vaccine (AZD1222) against SARS-CoV-2: an interim analysis of four randomised controlled trials in Brazil, South Africa, and the UK. Lancet.

[5] Polack FP (2020). Safety and Efficacy of the BNT162b2 mRNA Covid-19 Vaccine. N. Engl. J. Med.

[6] Ssentongo P (2022). SARS-CoV-2 vaccine effectiveness against infection, symptomatic and severe COVID-19: a systematic review and meta-analysis. BMC Infect. Dis..

[7] Patalon T (2022). Waning effectiveness of the third dose of the BNT162b2 mRNA COVID-19 vaccine. Nat. Commun..

[8] Rennert L, Ma Z, McMahan CS, Dean D (2022). Effectiveness and protection duration of Covid-19 vaccines and previous infection against any SARS-CoV-2 infection in young adults. Nat. Commun..

[9] Islam N, Sheils NE, Jarvis MS, Cohen K (2022). Comparative effectiveness over time of the mRNA-1273 (Moderna) vaccine and the BNT162b2 (Pfizer-BioNTech) vaccine. Nat. Commun..

[10] Azzi L (2022). Mucosal immune response in BNT162b2 COVID-19 vaccine recipients. EBioMedicine.

[11] Tang J (2022). Respiratory mucosal immunity against SARS-CoV-2 following mRNA vaccination. Sci. Immunol.

[12] Cele S (2022). Omicron extensively but incompletely escapes Pfizer BNT162b2 neutralization. Nature.

[13] Garcia-Valtanen P (2022). SARS-CoV-2 Omicron variant escapes neutralizing antibodies and T cell responses more efficiently than other variants in mild COVID-19 convalescents. Cell Rep. Med..

[14] Cao Y (2022). BA.2.12.1, BA.4 and BA.5 escape antibodies elicited by Omicron infection. Nature.

[15] Altarawneh HN (2022). Protective Effect of Previous SARS-CoV-2 Infection against Omicron BA.4 and BA.5 Subvariants. N. Engl. J. Med.

[16] Lange J, Rivera-Ballesteros O, Buggert M (2022). Human mucosal tissue-resident memory T cells in health and disease. Mucosal Immunol..

[17] Clark RA (2015). Resident memory T cells in human health and disease. Sci. Transl. Med.

[18] Grau-Expósito J (2021). Peripheral and lung resident memory T cell responses against SARS-CoV-2. Nat. Commun..

[19] Nguyen TH (2021). Influenza, but not SARS-CoV-2, infection induces a rapid interferon response that wanes with age and diminished tissue-resident memory CD8(+) T cells. Clin. Transl. Immunol..

[20] Allen AC (2018). Sustained protective immunity against Bordetella pertussis nasal colonization by intranasal immunization with a vaccine-adjuvant combination that induces IL-17-secreting T(RM) cells. Mucosal Immunol..

[21] Wilk MM (2019). Immunization with whole cell but not acellular pertussis vaccines primes CD4 T(RM) cells that sustain protective immunity against nasal colonization with Bordetella pertussis. Emerg. Microbes Infect..

[22] Dunne A (2015). A novel TLR2 agonist from Bordetella pertussis is a potent adjuvant that promotes protective immunity with an acellular pertussis vaccine. Mucosal Immunol..

[23] Yin Q (2012). Cyclic di-GMP sensing via the innate immune signaling protein STING. Mol. Cell.

[24] Chasaide CN, Mills KHG (2020). Next-generation pertussis vaccines based on the induction of protective T cells in the respiratory tract. Vaccines (Basel).

[25] Wong TY (2022). Intranasal administration of BReC-CoV-2 COVID-19 vaccine protects K18-hACE2 mice against lethal SARS-CoV-2 challenge. NPJ Vaccines.

[26] Alu A (2022). Intranasal COVID-19 vaccines: from bench to bed. EBioMedicine.

[27] Lewis DJ (2009). Transient facial nerve paralysis (Bell’s palsy) following intranasal delivery of a genetically detoxified mutant of Escherichia coli heat labile toxin. PLoS ONE.

[28] Muruganandah V, Sathkumara HD, Navarro S, Kupz A (2018). A systematic review: the role of resident memory T cells in infectious diseases and their relevance for vaccine development. Front Immunol..

[29] Lucas C (2021). Delayed production of neutralizing antibodies correlates with fatal COVID-19. Nat. Med..

[30] Eisenbarth SC (2021). CD4(+) T cells that help B cells: a proposal for uniform nomenclature. Trends Immunol..

[31] Warfel JM, Zimmerman LI, Merkel TJ (2014). Acellular pertussis vaccines protect against disease but fail to prevent infection and transmission in a nonhuman primate model. Proc. Natl Acad. Sci. USA.

[32] Mills KH, Skehel JJ, Thomas DB (1986). Extensive diversity in the recognition of influenza virus hemagglutinin by murine T helper clones. J. Exp. Med..

[33] Mercado NB (2020). Single-shot Ad26 vaccine protects against SARS-CoV-2 in rhesus macaques. Nature.

[34] Vogel AB (2021). BNT162b vaccines protect rhesus macaques from SARS-CoV-2. Nature.

[35] van Doremalen N (2020). ChAdOx1 nCoV-19 vaccine prevents SARS-CoV-2 pneumonia in rhesus macaques. Nature.

[36] Corbett KS (2020). Evaluation of the mRNA-1273 vaccine against SARS-CoV-2 in nonhuman primates. N. Engl. J. Med..

[37] Finazzi S, Perego M, Tricella G, Poole D, Ranieri VM (2023). SARS-CoV-2 breakthrough infections in vaccinated individuals requiring ventilatory support for severe acute respiratory failure. Intensive Care Med.

[38] Barría MI (2013). Localized mucosal response to intranasal live attenuated influenza vaccine in adults. J. Infect. Dis..

[39] Sheikh-Mohamed S (2022). Systemic and mucosal IgA responses are variably induced in response to SARS-CoV-2 mRNA vaccination and are associated with protection against subsequent infection. Mucosal Immunol..

[40] Liew F (2023). SARS-CoV-2-specific nasal IgA wanes 9 months after hospitalisation with COVID-19 and is not induced by subsequent vaccination. EBioMedicine.

[41] Ghazanfar H (2022). Impact of COVID-19 on the gastrointestinal tract: a clinical review. Cureus.

[42] Teijaro JR (2011). Cutting edge: tissue-retentive lung memory CD4 T cells mediate optimal protection to respiratory virus infection. J. Immunol..

[43] Ellebedy AH, Webby RJ (2009). Influenza vaccines. Vaccine.

[44] Sridhar S, Brokstad KA, Cox RJ (2015). Influenza vaccination strategies: comparing inactivated and live attenuated influenza vaccines. Vaccines (Basel).

[45] Galli G (2009). Adjuvanted H5N1 vaccine induces early CD4+ T cell response that predicts long-term persistence of protective antibody levels. Proc. Natl Acad. Sci. USA.

[46] Magnusson SE (2018). Matrix-M™ adjuvant enhances immunogenicity of both protein- and modified vaccinia virus Ankara-based influenza vaccines in mice. Immunol. Res..

[47] Tian JH (2021). SARS-CoV-2 spike glycoprotein vaccine candidate NVX-CoV2373 immunogenicity in baboons and protection in mice. Nat. Commun..

[48] Richmond P (2021). Safety and immunogenicity of S-Trimer (SCB-2019), a protein subunit vaccine candidate for COVID-19 in healthy adults: a phase 1, randomised, double-blind, placebo-controlled trial. Lancet.

[49] Thuluva S (2022). Evaluation of safety and immunogenicity of receptor-binding domain-based COVID-19 vaccine (Corbevax) to select the optimum formulation in open-label, multicentre, and randomised phase-1/2 and phase-2 clinical trials. EBioMedicine.

[50] Gary EN (2022). Mucosal chemokine adjuvant enhances synDNA vaccine-mediated responses to SARS-CoV-2 and provides heterologous protection in vivo. Cell Rep. Med..

[51] Sato-Kaneko F (2022). A Dual Adjuvant System for Intranasal Boosting of Local and Systemic Immunity for Influenza Vaccination. Vaccines (Basel).

[52] Mao T (2022). Unadjuvanted intranasal spike vaccine elicits protective mucosal immunity against sarbecoviruses. Science.

[53] Madhavan M (2022). Tolerability and immunogenicity of an intranasally-administered adenovirus-vectored COVID-19 vaccine: An open-label partially-randomised ascending dose phase I trial. EBioMedicine.

[54] Wilson, H. L., Gerdts, V. & Babiuk, L. A. Mucosal vaccine development for veterinary and aquatic diseases. *Mucosal Vaccines*, Elsevier; 811–829 (2020).

[55] Gerdts V, Mutwiri GK, Tikoo SK, Babiuk LA (2006). Mucosal delivery of vaccines in domestic animals. Vet. Res.

[56] Carter NJ, Curran MP (2011). Live attenuated influenza vaccine (FluMist®; Fluenz™): a review of its use in the prevention of seasonal influenza in children and adults. Drugs.

[57] Lim JME (2022). SARS-CoV-2 breakthrough infection in vaccinees induces virus-specific nasal-resident CD8+ and CD4+ T cells of broad specificity. J. Exp. Med..

[58] Choi SJ (2022). T cell epitopes in SARS-CoV-2 proteins are substantially conserved in the Omicron variant. Cell Mol. Immunol..

[59] Sette A, Crotty S (2021). Adaptive immunity to SARS-CoV-2 and COVID-19. Cell.

[60] Syed AM (2022). Omicron mutations enhance infectivity and reduce antibody neutralization of SARS-CoV-2 virus-like particles. Proc. Natl Acad. Sci. USA.

[61] Abavisani M (2022). Mutations in SARS-CoV-2 structural proteins: a global analysis. Virol. J..

[62] Phelan T (2021). Dynamic assay for profiling anti-SARS-CoV-2 antibodies and their ACE2/Spike RBD neutralization capacity. Viruses.

