# Supplementary material for: Intranasal COVID-19 vaccine induces respiratory memory T cells and protects K18-hACE mice against SARS-CoV-2 infection
Source: NPJ Vaccines. 2023 May 13;8:68. doi: 10.1038/s41541-023-00665-3 (PMC10182552; doi:10.1038/s41541-023-00665-3)
Supplement: Supplementary file 1 — Supplementary Material [file 41541_2023_665_MOESM1_ESM.pdf]

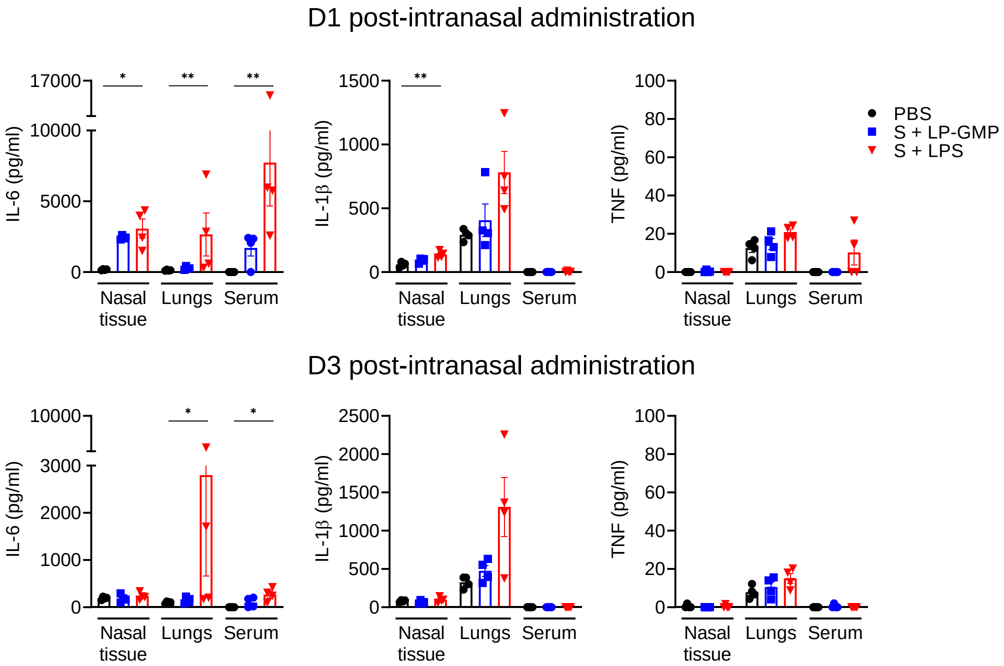

Supplementary Figure 1. Inflammatory responses induced with Spike-LP-GMP vaccine.

Mice were anesthetized with ketamine and xylazine and PBS, Spike-LP-GMP (3 µg of Spike trimer, 10 µg of C-di-GMP, 50 µg of LP1569), or Spike-LPS (3 µg of Spike trimer, 50 µg of LPS) was administered via intranasal route (n=4/group). Mice were euthanized 24 h or 72 h after administration. Serum, left lobe lung homogenate and nasal tissue homogenate were collected for analysis of inflammatory cytokines (IL-6, IL-1β, TNF) by ELISA. Data were analyzed by one-way ANOVAs followed by post hoc Tukey's test for multiple comparisons (*P < 0.05, **P < 0.01, ***P < 0.001, and ****P < 0.0001), error bars, SEM. Results are representative of two independent experiments.

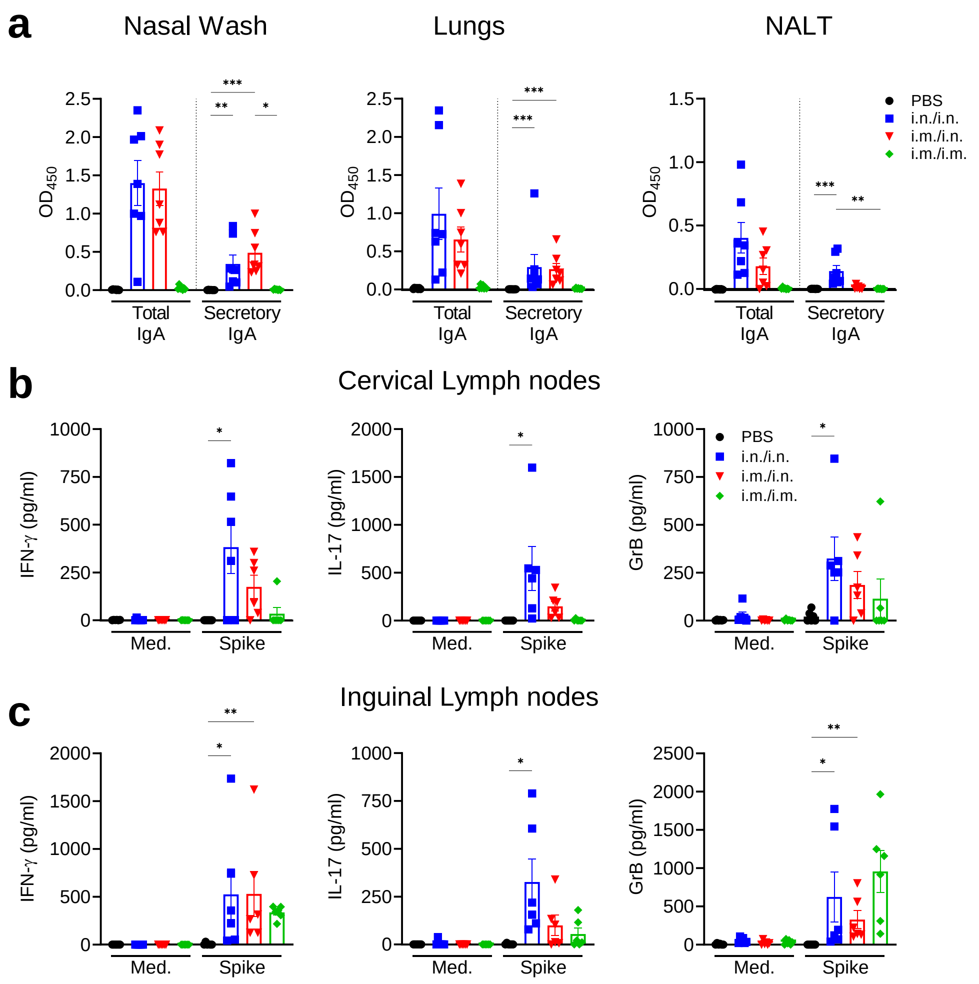

Supplementary Figure 2. Immunogenicity of intranasal administration of Spike-LP-GMP vaccine.

C57BL/6 mice (n=7/group) were immunized twice (0 and 21 days) i.n./i.n., i.m./i.n. or i.m./i.m. with the Spike-LP-GMP vaccine or PBS and euthanized on day 35 for samples collection. Lung, Nasal wash, NALT (nasal associated lymphoid tissue), cervical and inguinal lymph nodes were collected at day 35. (**a**) Spike-specific total IgA and Spike-specific secretory IgA were analyzed in the Nasal wash (1/4 dilution), (**b**) lung (1/20 dilution), and (**c**) NALT (1/2 dilution) by ELISA. Spike-specific IFN-γ, IL-17 and GrB production by cervical LN cells (**b**) and inguinal LN cells (**c**) stimulated for 72h with medium alone or Spike trimer (2.5 µg/ml), were quantified by ELISA. Data were analyzed by one-way ANOVAs followed by post hoc Tukey's test for multiple comparisons (*P < 0.05, **P < 0.01, ***P < 0.001, and ****P < 0.0001), error bars, SEM. Results are representative of two independent experiments.

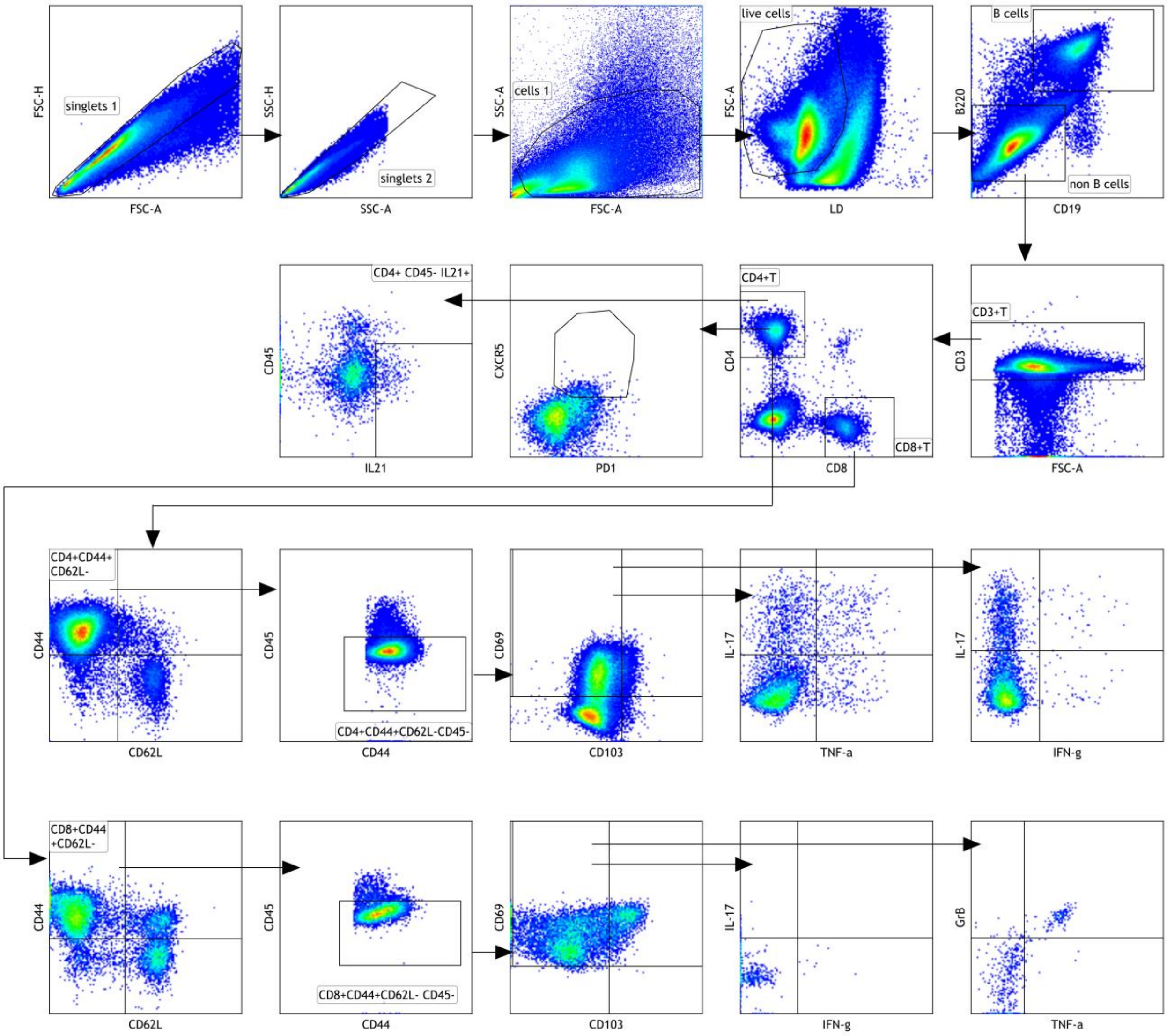

Supplementary Figure 3. Flow cytometry gating strategy used for lung and nasal tissue cells analyses.

Singlets were gated on FSC-H/FSC-A and then SSC-H/SSC-A. Cells were gated on SSC-A/FSC-A. Live cells were gated on FSC-A/Live Dead$^-$. Non-B cells were gated on B220$^-$/CD19$^-$. CD3$^+$ T cells were gated on CD3$^+$/FSC-A. CD4$^+$ and CD8$^+$ T cells were gated on CD4$^+$/CD8$^+$. then out of the CD4$^+$ T cells, CD4$^+$ T_{FH} were gated on PD-1$^+$/CXCR-5$^+$. Out of CD4$^+$ T cells, resident CD4$^+$ T cells secreting IL-21 were gated on CD45$^-$/IL-21$^+$. Out of CD4$^+$ T cells, effector memory CD4$^+$ T cells were gated on CD44$^+$/CD62L$^-$.

Out of CD4$^+$ CD44$^+$ CD62L$^-$ T cells, resident CD4$^+$ CD44$^+$ CD62L$^-$ were gated on CD45$^-$/CD44$^+$. Out of CD4$^+$ CD44$^+$ CD62L$^-$ CD45$^-$ T cells, resident memory CD4$^+$ T cells were gated on CD69$^+$/CD103$^+$. Out of CD4$^+$ CD44$^+$ CD62L$^-$ CD45$^-$ CD69$^+$ T cells, resident memory CD4$^+$ T cells secreting cytokines were gated on IL-17$^+$/TNF$^+$ and IL-17$^+$/IFN-γ^+.

Out of CD8$^+$ T cells, effector memory CD8$^+$ T cells were gated on CD44$^+$/CD62L$^-$. Out of CD8$^+$ CD44$^+$ CD62L$^-$ T cells, resident CD8$^+$ CD44$^+$ CD62L$^-$ were gated on CD45$^-$/CD44$^+$. Out of CD8$^+$ CD44$^+$ CD62L$^-$ CD45$^-$ T cells, resident memory CD8$^+$ T cells were gated on CD69$^+$/CD103$^+$. Out of CD8$^+$ CD44$^+$ CD62L$^-$ CD45$^-$ CD69$^+$ T cells, resident memory CD8$^+$ T cells secreting cytokines were gated on GrB$^+$/TNF$^+$ and IL-17$^+$/IFN-γ^+. Flow cytometry Data were analyzed using Kaluza Analysis Software (Beckman Coulter).

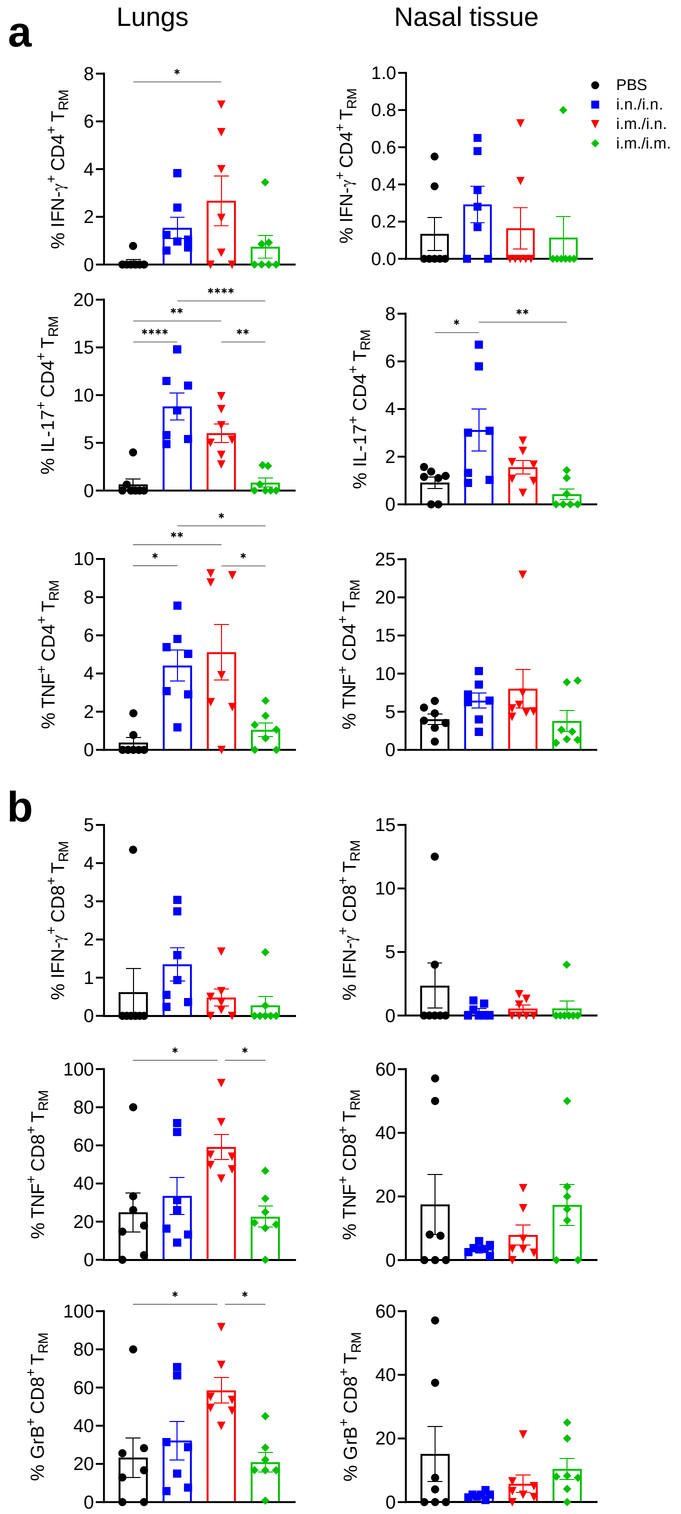

Supplementary Figure 4. Frequency of Spike-specific cytokine-secreting CD4+ T_{RM} and CD8+ T_{RM} cells.

C57BL/6 mice (n=7/group) were immunized twice (0 and 21 days) i.n./i.n., i.m./i.n. or i.m./i.m. with the Spike-LP-GMP vaccine or PBS and euthanized on day 35 for samples collection. Lung and Nasal tissue were collected at day 35 and stimulated for 18 h with Spike trimer (2.5 µg/ml). Cells were analyzed using flow cytometry. (**a**) Percentage of resident CD4+ T_{RM} cells (CD4+ CD44+ CD62L- CD45- CD69+) secreting IFN-γ, IL-17 and TNF in the lung and nasal tissue. (**b**) Percentage of resident CD8+ T_{RM} cells (CD8+ CD44+ CD62L- CD45- CD69+) secreting IFN-γ, TNF and GrB in the lung and nasal tissue. Data were analyzed by one-way ANOVAs followed by post hoc Tukey's test for multiple comparisons (*P < 0.05, **P < 0.01, ***P < 0.001, and ****P < 0.0001), error bars, SEM. Results are representative of three independent experiments.

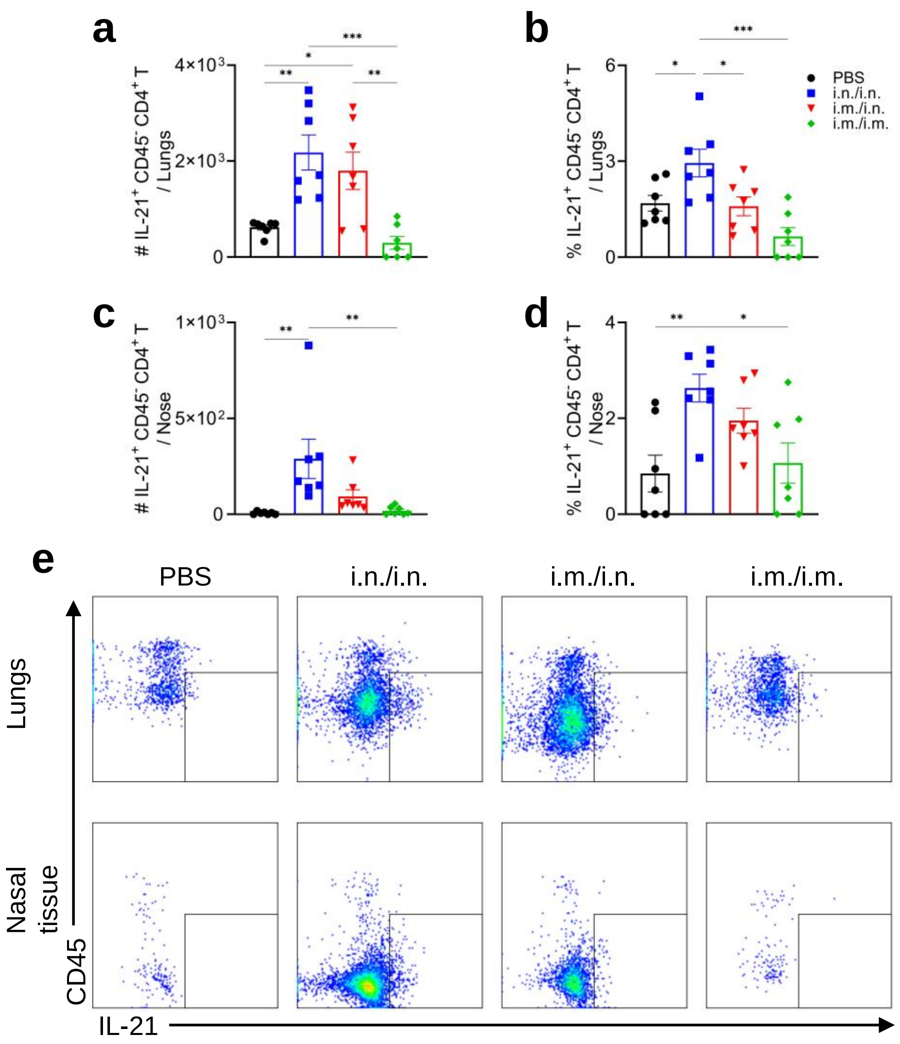

Supplementary Figure 5. IL-21 secreting Spike-specific resident CD4+ T cells in the lung and nasal tissue of immunized mice.

C57BL/6 mice (n=7/group) were immunized twice (0 and 21 days) i.n./i.n., i.m./i.n. or i.m./i.m. with the Spike-LP-GMP vaccine or PBS and euthanized on day 35 for samples collection. Lung and Nasal tissue were collected at day 35 and stimulated for 18 h with Spike trimer (2.5 µg/ml). Cells were analyzed using flow cytometry. (**a**) Number of resident CD4+ T cells secreting IL-21 in the lung. (**b**) Percentage of resident CD4+ T cells secreting IL-21 in the lung. (**c**) Number of resident CD4+ T cells secreting IL-21 in the nasal tissue, (**d**) Percentage of resident CD4+ T cells secreting IL-21 in the nasal tissue. (**e**) Representative flow cytometry plot of resident CD4+ T cells secreting IL-21 in the lung and nasal tissue. Data were analyzed by one-way ANOVAs followed by post hoc Tukey's test for multiple comparisons (*P < 0.05, **P < 0.01, ***P < 0.001, and ****P < 0.0001), error bars, SEM.

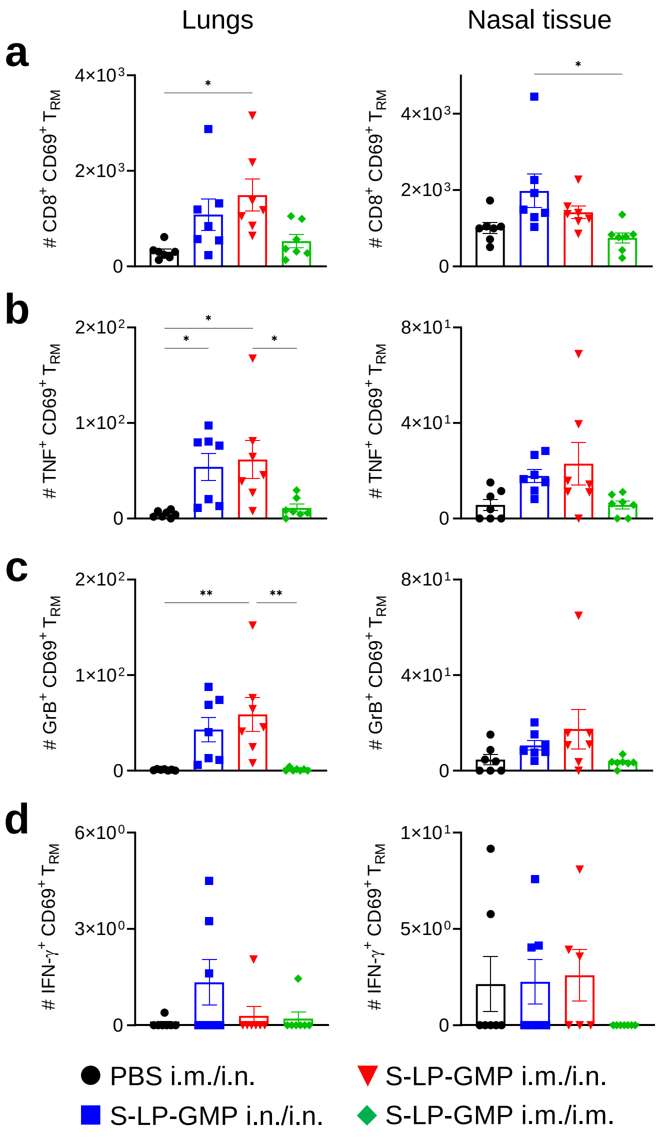

Supplementary Figure 6. Spike-specific CD8$^+$ T$_{RM}$ cells persist in the lungs and nasal tissue for at least 3 months post immunization. Mice were immunized as described in Fig. 3, lung and nasal tissue samples were collected on day 111. **a** Number of CD8$^+$ T$_{RM}$ cells (CD44$^+$CD62L$^-$CD45$^-$CD69$^+$) in the lung and nasal tissue. **b-c** Number of Spike-specific CD8$^+$ T$_{RM}$ cells secreting TNF (**b**), GrB (**c**) or IFN-γ (**d**) in the lung and nasal tissue following 18 h stimulation with Spike trimer (2.5 μg/ml). Data were analyzed by one-way ANOVAs followed by post hoc Tukey's test for multiple comparisons (*$P < 0.05$, **$P < 0.01$, ***$P < 0.001$, and ****$P < 0.0001$), error bars, SEM.

Supplementary Table 1. List of antibodies

Unsimulated cells

	marker	flurochrome	dilution	clone	reference	supplier
**Extracellular staining**	Live dead	AQUA	1/600	L34957	L34957	BD Bioscience
	CXCR-5	Biotin/APC	1/200	L138D7	13-7185-82	Biolegend
	CD69	AF647	1/200	H1.2F3	104518	Biolegend
	CD8	AF700	1/200	53-6.7	56-0081-82	invitrogen/ebioscience
	CD3	EF450	1/200	145-2C11	48-0031-82	ebioscience
	PD-1	BV711	1/200	29F.1A12	135231	Biolegend
	CD4	BV785	1/200	RM4-5	100552	Biolegend
	CD103	PE-CF594	1/200	M290	565849	BD Bioscience
	B220	PECY5	1/200	RA3-6B2	565849	Biolegend
	CD62L	PECY7	1/200	MEL-14	565849	Biolegend
	CD19	BB700	1/200	1D3	566412	BD Biosciences
	CD44	AF594	1/200	IM7	103054	Biolegend
	Fc Block		1/200	2.4G2	553142	BD Biosciences

**intravenous injection**	CD45	PE	3.75µg in 200µL	30-F11		invitrogen/ebioscience

Antigen-stimulated cells

	marker	flurochrome	dilution	clone	reference	supplier
**Extracellular staining**	Life dead	AQUA	1/600	L34957	L34957	BD Bioscience
	CD69	AF647	1/200	H1.2F3	104518	Biolegend
	CD8	AF700	1/200	53-6.7	56-0081-82	invitrogen/ebioscience
	CD4	BV785	1/200	RM4-5	100552	Biolegend
	CD3	APC-EF780	1/200	145-2C11	47003182	invitrogen/ebioscience
	CD103	PE-CF594	1/200	M290	565849	BD Bioscience
	CD62L	PE-Cy5	1/100	MEL-14	565849	Biolegend
	CD44	AF594	1/200	IM7	103054	Biolegend
	CD19	PE-Cy7	1/200	1D3	115520	ebioscience
	Fc Block 2.4G2		1/200	2.4G2	553142	BD Biosciences

**Intracellular staining**	IL-17	V450	1/200	TC11-18H10	560522	BD Bioscience
	IFN-g	APC	1/200	XMG1.2	MABF1516	invitrogen/ebioscience
	TNF	BV650	1/200	MP6-XT22	563943	BD Bioscience
	granzyme B	FITC	1/200	NGZB	563943	invitrogen/ebioscience
	IL-21	PE	1/200	mhalx21	12-7213-82	BD Bioscience

Supplementary Table 2. List of reagents

	Name	Reference	supplier	
**ELISA Kits**	IL-17	DY421	Bio-techne	
	Granzyme B	DY1865-05	Bio-techne	
	TNF	DY410	Bio-techne	
	IL-1β	DY401	Bio-techne	
	IFN-γ	551216/554410	BD	
	IL-6	554400/554402	BD	
**ELISA antibodies**	Goat anti-mouse IgG	G-21234	Biosciences	
	Goat anti-mouse IgG2c	STAR135P	Accuscience	
	Goat anti-mouse IgA	1040-05	Southernbiotech	
	Anti-mouse secretory IgA component	RE03MOES01461	AssayGenie - MSC	
**MSD kit**	Meso-scale discovery SARS-CoV-2/ACE-2 neutralization assay	K15386U-2	Meso Scale Diagnostics	
**Molecules**	Spike trimer wuhan-1	Custom made	Peak protein	UK
	Spike trimer β strain	Custom made	Peak protein	UK
	LP1569	Custom made	EMC microcollections	Germany
	C-di-GMP	tlrl-nacdg-5	Invivogen	France
	Human ACE-2	10108-H08H-B	Sino Biological	China
**Softwares**	Prism 9		Graph-Pad	
	Kaluza		Beckman Coulter	
	Softmax Pro 7.0.3		Molecular Devices	
	SpectroFlo		Cytec Biosciences	
